# Exploring the cross‐cancer effect of smoking and its fingerprints in blood DNA methylation on multiple cancers: A Mendelian randomization study

**DOI:** 10.1002/ijc.34656

**Published:** 2023-07-14

**Authors:** Yajing Zhou, Xuan Zhou, Jing Sun, Lijuan Wang, Jianhui Zhao, Jie Chen, Shuai Yuan, Yazhou He, Maria Timofeeva, Athina Spiliopoulou, Ines Mesa‐Eguiagaray, Susan M. Farrington, Kefeng Ding, Malcolm G Dunlop, Xiao Qian, Evropi Theodoratou, Xue Li

**Affiliations:** ^1^ Colorectal Surgery and Oncology, Key Laboratory of Cancer Prevention and Intervention, Ministry of Education, The Second Affiliated Hospital Zhejiang University School of Medicine Hangzhou China; ^2^ Department of Big Data in Health Science, School of Public Health and The Second Affiliated Hospital Zhejiang University School of Medicine Hangzhou China; ^3^ Centre for Population Health Sciences, Usher Institute University of Edinburgh Edinburgh UK; ^4^ Centre for Global Health Sciences, Usher Institute University of Edinburgh Edinburgh UK; ^5^ Unit of Cardiovascular and Nutritional Epidemiology Institute of Environmental Medicine, Karolinska Institutet Stockholm Sweden; ^6^ Department of Oncology, West China School of Public Health and West China Fourth Hospital Sichuan University Chengdu China; ^7^ Danish Institute for Advanced Study (DIAS), Epidemiology, Biostatistics and Biodemography Research Unit Institute of Public Health, University of Southern Denmark Odense Denmark; ^8^ Cancer Research UK Edinburgh Centre, Medical Research Council Institute of Genetics and Cancer University of Edinburgh Edinburgh UK; ^9^ Colon Cancer Genetics Group, Institute of Genetics and Cancer University of Edinburgh Edinburgh UK

**Keywords:** cross‐cancer effect, DNA methylation, Mendelian randomization, methylation quantitative trait loci, smoking

## Abstract

Aberrant smoking‐related DNA methylation has been widely investigated as a carcinogenesis mechanism, but whether the cross‐cancer epigenetic pathways exist remains unclear. We conducted two‐sample Mendelian randomization (MR) analyses respectively on smoking behaviors (age of smoking initiation, smoking initiation, smoking cessation, and lifetime smoking index [LSI]) and smoking‐related DNA methylation to investigate their effect on 15 site‐specific cancers, based on a genome‐wide association study (GWAS) of 1.2 million European individuals and an epigenome‐WAS (EWAS) of 5907 blood samples of Europeans for smoking and 15 GWASs of European ancestry for multiple site‐specific cancers. Significantly identified CpG sites were further used for colocalization analysis, and those with cross‐cancer effect were validated by overlapping with tissue‐specific eQTLs. In the genomic MR, smoking measurements of smoking initiation, smoking cessation and LSI were suggested to be casually associated with risk of seven types of site‐specific cancers, among which cancers at lung, cervix and colorectum were provided with strong evidence. In the epigenetic MR, methylation at 75 CpG sites were reported to be significantly associated with increased risks of multiple cancers. Eight out of 75 CpG sites were observed with cross‐cancer effect, among which cg06639488 (EFNA1), cg12101586 (CYP1A1) and cg14142171 (HLA‐L) were validated by eQTLs at specific cancer sites, and cg07932199 (ATXN2) had strong evidence to be associated with cancers of lung (coefficient, 0.65, 95% confidence interval [CI], 0.31‐1.00), colorectum (0.90 [0.61, 1.18]), breast (0.31 [0.20, 0.43]) and endometrium (0.98 [0.68, 1.27]). These findings highlight the potential practices targeting DNA methylation‐involved cross‐cancer pathways.

## Graphical abstract

What's new?

Despite that smoking has been widely considered as a risk factor for multiple cancers, the smoking‐related cross‐cancer epigenetic pathway remains underexplored. In our study, the investigation of the genetic and epigenetic association between smoking and 15 site‐specific cancers revealed the mediating effect of smoking‐related aberrant methylation of CpG sites of cg06639488 (EFNA1), cg12101586 (CYP1A1), cg14142171 (HLA‐L) and cg07932199 (ATXN2) on increased risks of lung, colorectal, breast, cervix cancers, highlighting underlying epigenetic pathways of carcinogenesis.

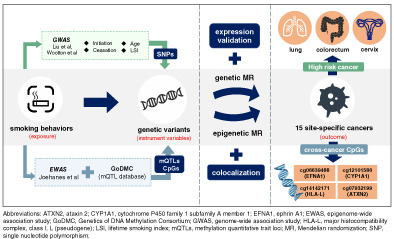

**Abbreviations**
BCAC: the Breast Cancer Association Consortium
CI: confidence interval
CpG: cytosine‐phosphate‐guanine
CRC: colorectal cancer
E2C2: the Epidemiology of Endometrial Cancer Consortium
ECAC: the Endometrial Cancer Association Consortium
FDR: false discovery rate
GoDMC: Genetics of DNA Methylation Consortium
GTEx: the Genotype‐Tissue Expression resource
ILCCO: the International Lung Cancer Consortium
IV: instrumental variant
IVW: inverse‐variance weighted
LD: linkage disequilibrium
LSI: lifetime smoking index
MHC: major histocompatibility complex
MR: Mendelian randomization
OCAC: the Ovarian Cancer Association Consortium
OR: odds ratios
PPA: posterior probability of association
QTLs: quantitative trait loci
SNP: single nucleotide polymorphism

## 1 INTRODUCTION

Smoking has been widely recognized as a risk factor for numerous diseases including cancer. Observational And experimental studies have confirmed the causal relationship between smoking and the risk of lung cancer,^1^, ^2^ and other common cancers like breast cancer,^3^ prostate cancer,^4^ ovarian cancer and cervix cancer have also been deemed as potentially consequential events associated with smoking.^5^

Studies have found aberrant DNA methylation can be induced by smoking behaviors,^6^ which is considered as a potential mechanism to trigger carcinogenesis. Also, hypotheses of DNA methylation‐related epigenetic modification have long been investigated and widely elaborated as one of the mechanisms of carcinogenesis.^7^ DNA methylation is classically characterized by the process of forming the 5‐methylcytosine in the C5 position of cytosine‐phosphate‐guanine (CpG) dinucleotides. This is prone to obstacle the combination of transcription complex and DNA and cause nonprogrammed alteration in downstream gene expression,^8^ such as hypomethylation in activating proto‐oncogenes and hypermethylation in the silencing of tumor‐suppressor genes in the promoter region^9^ in carcinoma tissues. A number of studies have investigated the association of smoking and single site‐specific cancer via differentiated methylation level^10^, ^11^, ^12^ suggesting the key role of methylation in the process of carcinogenesis. However, whether the epigenetic effect is exerted universally across multiple cancers remains unknown, and whether the mechanisms of methylation are shared through some specific CpG sites or common pathways is worth exploring.

Mendelian randomization analysis is a method that uses genetic variants, for example, single nucleotide polymorphisms (SNPs) or quantitative trait loci (QTLs) as proxies for risk factors of interest to explore the causality between exposure and disease.^13^ This minimizes the unmeasured confounding effects and diminishes reverse causality. In our study, we sequentially conducted two two‐sample MR analyses using instrumental variants (IVs) of SNPs as genetic proxies and methylation QTLs (mQTLs) as epigenetic proxies of methylation at CpG sites to explore the causal effect of smoking on the risk of multiple site‐specific cancers. We further assessed the cross‐cancer effects of smoking‐related blood methylation and validated the tissue‐specificity with expression‐QTLs (eQTLs).

## 2 MATERIALS AND METHODS

### 2.1 Study design

**FIGURE 1:**
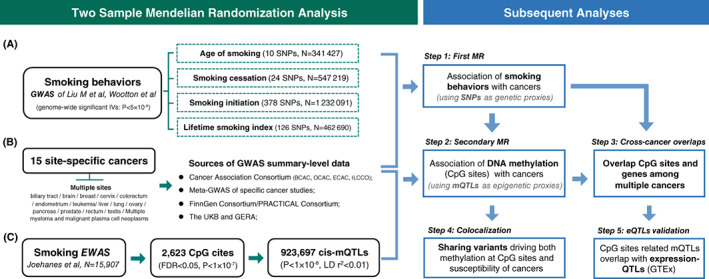
Schematic diagram of the study design. EWAS, epigenome‐wide association study; GWAS, genome‐wide association study; mQTLs, methylation quantitative trait loci; MR, Mendelian randomization; SNP, single nucleotide polymorphism.

In our study, we firstly conducted a two‐sample Mendelian randomization (MR) analysis to investigate the causal effect of smoking on genetic predisposition to 15 site‐specific cancers, in which we chose three phenotypes of smoking behaviors (age of initiation, smoking initiation and smoking cessation) and an aggregative lifetime smoking index (LSI) as specific measurements of smoking. Then we performed a second two‐sample MR analysis to reveal the aforementioned causality on the epigenome‐wide level, using mQTLs as IVs for smoking‐related blood DNA methylation at CpG sites, and further focused on those that have a cross‐cancer effect and are validated with tissue‐specific eQTLs. For the significant CpG cites, we also conducted a colocalization analysis to investigate the effect of sharing variants both on DNA methylation and susceptibility of cancers (Figure 1).

### 2.2 Data sources

#### 2.2.1 Genome‐wide association study summary‐level data of smoking behaviors

The IVs for four smoking behaviors were extracted from two genome‐wide association studies (GWASs) separately. First, SNPs associated with age of smoking initiation (10 associated variants, *N* = 341 427), smoking initiation (378 variants, *N* = 1 232 091), smoking cessation (24 variants, *N* = 547 219) at the significant threshold (*P* < 5 × 10^−8^) as genetic instruments were obtained from a published GWAS that identified variants associated with different aspects of smoking (initiation, cessation and heaviness) from a total of 1 232 091 individuals of European ancestry.^14^ In our study, smoking initiation and cessation are both binary phenotypes comparing individuals' smoking status, with current or previous smokers coded as “2” and never smokers coded as “1” for smoking initiation, and current smokers coded as “2” and previous smokers coded as “1” for smoking cessation. Then we derived genetic variants for LSI, an aggregative indicator of smoking, from a GWAS involving 462 690 individuals of European ancestry (126 independent, genome‐wide significant SNPs).^15^ Linkage disequilibrium (LD) was calculated based on 1000 Genomes European reference panel, and only genetic variants without LD (*r* ^2^ ≤ 0.01 and clump window >10 000 kb) were selected (Tables S1 and S2).

#### 2.2.2 Epigenome‐wide data of smoking‐related DNA methylation

The information of the association between smoking and DNA methylation (ie, smoking‐related CpG sites) was derived from a genome‐wide meta‐analysis measuring 5907 blood‐derived DNA samples from participants in 16 cohorts of the Cohorts for Heart and Aging Research in the Genetic Epidemiology Consortium.^6^ A total of 2623 CpG sites with differentiated methylation was identified between current smokers and never smokers (false discovery rate [FDR] < 0.05, *P* < 1 × 10^−7^). We then obtained CpG‐associated mQTLs as genetic proxies from Genetics of DNA Methylation Consortium (GoDMC) (http://mqtldb.godmc.org.uk/), a mQTL database containing genetic and methylation data from over 30 000 participants.^16^ DNA methylation data were quantified from bisulfite‐converted genomic blood DNA using Illumina Infinium HumanMethylation450 (HM450) BeadChip.

#### 2.2.3 GWAS summary‐level data of 15 site‐specific cancers

We obtained summary‐level data of 15 site‐specific cancers from publicly available datasets (Table S1): (1) endometrial cancer GWAS data were acquired from a meta‐GWAS of 17 cohort studies (via the Endometrial Cancer Association Consortium [ECAC], the Epidemiology of Endometrial Cancer Consortium [E2C2] and the UK Biobank) with 12 906 cancer cases and 108 979 country‐matched controls^17^; (2) colorectal cancer (CRC) GWAS data were acquired from a meta‐GWAS of 11 cohort studies of colorectal cancer with 16 871 cases and 26 328 controls^18^; (3) prostate cancer GWAS data were obtained from a meta‐analysis of 8 GWAS from PRACTICAL Consortium with 79 148 prostate cancer cases and 61 112 controls^19^; (4) GWAS of breast cancer (122 977 cases vs 105 974 controls) were obtained from the Breast Cancer Association Consortium (BCAC)^20^; (5) ovarian cancer (25 509 cases vs 40 941 controls) were derived from the Ovarian Cancer Association Consortium (OCAC)^21^; (6) lung cancer (11 348 cases vs 15 861 controls) were acquired from the International Lung Cancer Consortium (ILCCO)^22^; (7) GWAS data of liver cancer (304 cases vs 218 488 controls), brain cancer (464 cases vs 218 328 controls), biliary cancer (109 cases vs 174 006 controls), leukemia (663 cases vs 218 129 controls), testis cancer (199 cases vs 95 014 controls) and multiple myeloma and malignant plasma cell neoplasms (598 cases vs 218 194 controls) were acquired from the Finngen Consortium^23^; (8) GWAS of cervix cancer (6563 cases vs 410 350 controls) and pancreatic cancer (663 cases vs 410 350 controls) were obtained from The UK Biobank^24^ and GERA data.^25^ All acquired data were constricted to European ancestry.

### 2.3 Two‐sample MR

We successively performed two two‐sample MR analyses on the levels of genome and epigenome. In our first MR analysis, genetic variants (SNPs) identified for four measurements of smoking were employed as exposure to investigate its causal effect on the risk of multiple cancers. In the second phase, we identified mQTLs as epigenetic proxies for methylation at CpG cites associated with smoking. The effect allele of each mQTL was unified to be in the same direction with the effect of smoking on DNA methylation. We used Wald ratio to estimate the association when exposure had only one SNP for proxy, and inverse‐variance weighted (IVW) method with random‐effects to measure the combined effect for each exposure as the main method. Sensitivity analyses were additionally applied to improve the robustness of the results. The MR Egger regression and the intercept test were utilized to detect and correct for horizontal pleiotropy.^26^ The Weighted Median method was used to provide consistent estimates when valid IVs weighed more than 50%.^27^ The MR‐PRESSO method was also employed to detect horizontal pleiotropy (global test), correct outliers by removing them (outlier test) and assess its distortion significance (distortion test).^28^ The Cochrane's *Q* value was used to evaluate the heterogeneity of genetic variants (*Q* < 0.05). *F*‐statistics were calculated to measure the strength of instruments (*F* < 10 was considered to be a weak instrument).^29^ The beta coefficient was calculated per SD for each genetic instrument, and odds ratios (ORs) with 95% confidence interval (CI) were scaled to per one SD increase in genetically predicted smoking and one‐unit increase in the log OR of liability to multiple cancers. False discovery rate was computed for multiple‐testing (FDR < 0.05). MR analyses were performed by using the “TwoSampleMR” R package.

For the significant CpG sites among multiple cancers in the second MR analysis, we further identified those that had cross‐cancer associations. The mQTLs of CpG sites with cross‐cancer effect were then obtained and searched in the Genotype‐Tissue Expression (GTEx) resource^30^ to further investigate their expression effect as eQTLs in the cancer‐associated tissues. The significance threshold of expression evidence was set by both *P*‐values (eQTL effect size) and *m*‐values (existence of eQTL‐effect in the specific tissue in the cross‐tissue meta‐analysis),^31^, ^32^ and mQTLs that met *P*‐value <.05/(the number of SNP‐gene pairs) after Bonferroni correction and *m*‐value >0.9 were indicated to have a statistically significant eQTL effect.

### 2.4 Colocalization analysis

Among the CpG sites that were significantly associated with risk of multiple cancers, we additionally performed a colocalization analysis to investigate whether the susceptibility to site‐specific cancers was driven by the same variants influencing methylation at the CpG sites. Observation of 75% or higher posterior probability of association (PPA) for both the summary effect of the CpG site and the single effect of a mQTL were deemed as evidence of colocalization. GWAS data for cancer and EWAS data for smoking‐methylation (with mQTLs as proxies) were the same as those used in the MR analysis. The colocalization analysis was performed by the “coloc” R package.^33^ All analyses were undertaken with R Software 4.2.1.

## 3 RESULTS

### 3.1 MR analysis of smoking behaviors and multiple cancers

Genetic variants for three smoking behaviors (age of smoking, smoking initiation and smoking cessation) and LSI are shown in Table S2, and the *F*‐statistic for each IV was above 10, suggesting there was no substantial weak instrument bias.

**FIGURE 2:**
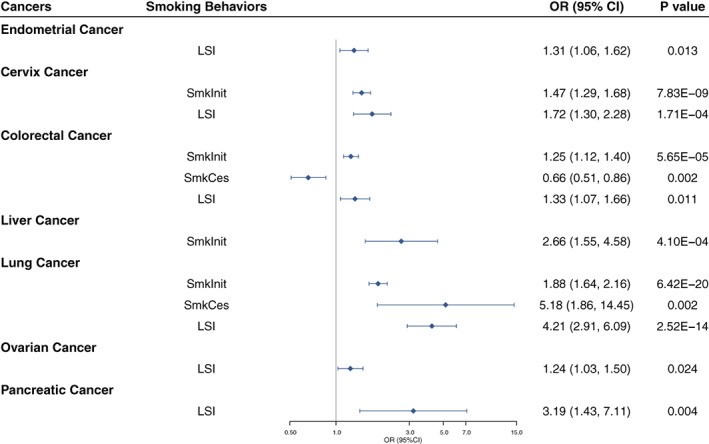
Forest plot of genetically predicted significant associations observed with smoking behaviors and multiple cancers. CI, confidence interval; LSI, lifetime smoking index; OR, odds ratio; SmkCes, smoking cessation; SmkInit, smoking initiation.

Seven types of site‐specific cancers were found significantly associated with three out of four measurements of smoking behaviors (except for age of smoking) utilizing the IVW method (Figure 2). The risk of lung cancer was indicated to be strongly affected by all three measurements of smoking: smoking initiation (OR, 1.88, 95%CI, 1.64‐2.16, FDR = 3.85 × 10^−18^), smoking cessation (OR, 5.86, 95%CI, 1.86‐14.45, FDR = 0.014) and LSI (OR, 4.21, 95%CI, 2.91‐6.09, FDR = 7.56 × 10^−13^). Increased risk of CRC was also reported by smoking initiation (OR, 1.25, 95%CI, 1.12‐1.40, FDR = 8.48 × 10^−4^), LSI (OR, 1.33, 95%CI, 1.07‐1.66, *P* = .011) and smoking cessation (OR, 0.66, 95%CI, 0.51‐0.86, FDR = 0.015). Cervix cancer was also suggested to be causally affected by smoking initiation (OR, 1.47, 95%CI, 1.29‐1.68, FDR = 1.57 × 10^−7^) and LSI (OR, 1.72, 95%CI, 1.30‐2.28, FDR = 0.002). Other cancers at specific sites of liver, endometrium, pancreas and ovary were also indicated to be consequently associated with smoking initiation or LSI. MR‐Egger and the intercept test found horizontal pleiotropy in the analyses of some smoking behaviors with cancers (eg, smoking initiation with CRC and lung cancer), and the MR‐PRESSO method indicated some outliers in the analyses (Table S3).

### 3.2 MR analysis of smoking‐related DNA methylation and multiple cancers

After FDR correction, 12 out of 15 specific cancers were observed to be associated with smoking‐related DNA methylation with more than one mQTLs for proxies (Table 1), among which breast cancer (33 CpG sites) and prostate cancer (27 CpG sites) ranked the highest in terms of the number of CpG sites (Table S4).

**TABLE 1:** Overview of CpG sites found in the second Mendelian randomization of smoking‐related DNA methylation and cancers.

Category of cancer	Number of CpG sites		
	With available mQTLs	*P* < .05	FDR < 0.05
Biliary cancer	1956	73	2
Brain cancer	1956	109	1
Breast cancer	1950	223	57
Cervix cancer	1954	110	6
Colorectal cancer	1956	157	6
Endometrial cancer	1954	140	9
Leukemia	1956	96	0
Liver cancer	1956	90	2
Lung cancer	1938	130	10
Multiple myeloma and malignant plasma cell neoplasms	1956	101	0
Ovarian cancer	1872	148	7
Pancreatic cancer	1954	78	0
Prostate cancer	1415	199	52
Rectum cancer	1955	118	1
Testis cancer	1956	109	2

Abbreviations: FDR, false discovery rate; mQTLs, methylation quantitative trait loci.

A total of 75 CpG sites was found to have significant effect on the risk of 10 site‐specific cancers, among which 8 CpG sites were observed to have cross‐cancer effect: cg02405476 (*UBE2C*), cg06639488 (*EFNA1*), cg07932199 (*ATXN2*), cg11152384 (*RP11‐554A11.8*), cg12101586 (*CYP1A1*), cg14142171 (*HLA‐L*), cg22533573 (*WT1*) and cg25727671 (*HOXA7*; Table 2 and Figure S1). Most of the CpG sites have a constricted influence on the onset risk with only two types of cancer, whereas cg07932199 was associated with four cancers: breast cancer (MR estimate, 0.31, 95%CI, 0.20‐0.43), colorectal cancer (MR estimate, 0.90, 95%CI, 0.61‐1.18), endometrial cancer (MR estimate, 0.98, 95%CI, 0.68‐1.27) and lung cancer (MR estimate, 0.65, 95%CI, 0.31‐1.00). After searching the GTEx project, 4 mQTLs of 3 CpG sites with cross‐cancer effect were provided with evidence of functioning as eQTLs (*P* < .05) of the same specific cancer tissues as reported in the second MR analysis. rs9330263 (cg06639488) was found acting as an eQTL of lung tissue (*P* = 2.10 × 10^−5^), and rs11264328 (cg06639488; *P* = 6.00 × 10^−3^), rs2472299 (cg12101586; *P* = 4.60 × 10^−5^) and rs1611463 (cg14142171; *P* = 5.00 × 10^6^) were all indicated as eQTLs of breast tissue (Table S5).

**TABLE 2:** Information of overlapped CpG cites among cancers.

CpG cites	Chr	Position	Gene‐symbol	Adjusted associations between CpG sites (mQTLs as proxies) with cancers				
				Cancer	SNP	Beta (95%CI) (adjusted)	*P*‐value	FDR
cg02405476	20	44 441 818	UBE2C	Breast cancer	2	0.05 (0.03, 0.08)	2.41E−04	0.018
cg02405476	20	44 441 818	UBE2C	Prostate cancer	2	0.07 (0.03, 0.10)	4.35E−04	0.014
cg06639488	1	155 103 222	EFNA1	Breast cancer	1	0.20 (0.12, 0.29)	2.48E−06	0.001
cg06639488	1	155 103 222	EFNA1	Lung cancer	1	0.52 (0.24, 0.79)	2.23E−04	0.018
cg07932199	12	112 008 034	ATXN2	Breast cancer	1	0.31 (0.20, 0.43)	1.52E−07	6.38E−05
cg07932199	12	112 008 034	ATXN2	Colorectal cancer	1	0.90 (0.61, 1.18)	7.01E−10	1.37E−06
cg07932199	12	112 008 034	ATXN2	Endometrial cancer	1	0.98 (0.68, 1.27)	1.27E−10	5.39E−08
cg07932199	12	112 008 034	ATXN2	Lung cancer	1	0.65 (0.31, 1.00)	1.95E−04	0.030
cg11152384	11	68 934 300	RP11‐554A11.8	Breast cancer	1	0.24 (0.12, 0.35)	5.66E−05	0.013
cg11152384	11	68 934 300	RP11‐554A11.8	Prostate cancer	1	0.95 (0.76, 1.14)	3.86E−22	9.15E−20
cg12101586	15	75 019 203	CYP1A1	Breast cancer	1	0.15 (0.09, 0.22)	6.66E−06	0.001
cg12101586	15	75 019 203	CYP1A1	Prostate cancer	1	0.14 (0.05, 0.23)	2.00E−03	0.044
cg14142171	6	30 228 101	HLA‐L	Breast cancer	5	0.06 (0.03, 0.09)	1.67E−04	0.014
cg14142171	6	30 228 101	HLA‐L	Cervix cancer	5	0.12 (0.05, 0.18)	5.63E−04	0.030
cg22533573	11	32 452 771	WT1	Ovarian cancer	2	0.39 (0.19, 0.59)	1.45E−04	0.023
cg22533573	11	32 452 771	WT1	Prostate cancer	2	0.23 (0.11, 0.35)	2.01E−04	0.013
cg25727671	7	27 193 351	HOXA7	Prostate cancer	1	0.23 (0.10, 0.37)	5.34E−04	0.024
cg25727671	7	27 193 351	HOXA7	Breast cancer	2	0.13 (0.06, 0.20)	3.35E−04	0.036

Abbreviations: Chr, chromosome; CI, confidence interval; FDR, false discovery rate; SNP, single nucleotide polymorphism.

### 3.3 Colocalization analysis

Among the 75 CpG sites, 15 were observed with evidence of colocalization within 6 site‐specific cancers, that is, breast cancer, colorectal cancer, endometrial cancer, liver cancer, lung cancer and prostate cancer. Especially, the methylation at CpG sites cg07932199 (*ATXN2*) was suggested with nearly 100% posterior probability of sharing causal variants (rs7310615 and rs3184504) with the susceptibility to four cancers: breast cancer, CRC, endometrial cancer and lung cancer. Six CpG sites (cg07932199, cg01765406, cg22561727, cg00867472, cg12593793 and cg26146569) only had less than 30 mQTLs available so they were excluded out of the colocalization analysis (Table S6 and Figures S2–S9).

## 4 DISCUSSION

In our study, we sequentially performed two two‐sample MR analyses investigating the association between smoking and multiple cancers on the genome‐ and epigenome‐wide level, and further validated the results by tissue‐specific eQTLs and colocalization analysis. The first MR suggested that smoking measurements of smoking initiation, smoking cessation and LSI were causally associated with the risk of seven site‐specific cancers, in which lung, colorectal and cervix cancer were strongly indicated. The second MR revealed the effect of blood DNA methylation at CpG sites on 12 cancers, in which 8 CpG sites were observed to have cross‐cancer effects. mQTLs of CpG sites cg06639488 (*EFNA1*), cg12101586 (*CYP1A1*) and cg14142171 (*HLA‐L*) was validated by eQTLs at specific cancer tissues, and cg07932199 (*ATXN2*) provided colocalization evidence of both methylation and susceptibility to multiple cancers.

Our first MR analysis implied that smoking behaviors were causally associated with cancers at seven specific sites, including cervix, colorectum, endometrium, liver, lung, pancreas and ovary, among which lung cancer, cervix cancer and CRC were strongly indicated. The association of smoking and cancers have been investigated widely in a vast number of observational and experimental studies,^5^, ^34^ revealing underlying mechanisms of carcinogens interacting with body vital components, bioactive substances and genetic environment.^35^, ^36^ For instance, studies investigating causal effects of several smoking behaviors observationally or experimentally suggested the same findings as ours on lung cancer,^1^, ^2^ cervix cancer^34^ and CRC.^37^ Notably, our results showed that smoking cessation was significantly associated with an increased risk of CRC (OR < 1 indicating risk effect). Given that our results of smoking initiation (comparing current/former smokers with nonsmokers) indicated the overall association of smoking with increased risk of CRC, which was also supported by subsequent epigenetic MR analysis, this finding did not affect our overall conclusions. Nonetheless, some previous studies have also implied similar clues for the contradictory findings. The genetic correlation analysis in the GWAS where we obtained three smoking measurements suggested that smoking cessation was negatively (with current smokers coded as “2” and previous smokers coded as “1”) associated with inflammatory bowel disease (especially ulcerative colitis), potentially indicating an irregular association pattern between smoking cessation and intestinal diseases.^14^ Nevertheless, since the relationships between smoking behaviors and cancers were complicated and the effect of smoking cessation was affected by other factors including smoking duration, smoking intensity, the age of quitting smoking and so forth,^38^ further evidence is needed.

A vast number of studies have revealed the effect of smoking exerted on DNA methylation across the whole epigenome,^6^ which is also responsible for increasing risk of multiple cancers. Our study found that the CpG site cg06639488 (*EFNA1*) to have cross‐cancer effect on breast and lung cancer, consistent with previous findings. EFNA1 belongs to the subfamily of ephrins acting as the ligands for Eph receptors, and the interaction of EFNA1 with its most common receptor EphA2 is deemed crucial to the onset of malignant tumors, possibly via regulation of cell cytoskeleton and cell adhesion.^39^, ^40^ The upregulation of EFNA1 has already been reported in a broad variety of cancers, for instance, a study has reported a higher transcription and expression of EFNA1 in breast cancer tissues than para‐cancerous tissues using the UALCAN database, elucidating the potential values of EPHA/EFNA family‐related pathways in predicting breast cancer.^41^ In addition, EphA2 was found overexpressed in diverse cancers, among which lung cancer, also reported in our findings, was provided with a pointed strategy targeting EPHA2 blockade.^42^ Given that EFNA1‐related pathways were widely observed in the pathogenesis of multiple cancers, and with a novel epigenetic perspective provided by our study that EFNA1 could possibly increase the risk of cancers through DNA methylation modification, the further mechanisms of the interaction between EFNA1 and cancers need to be investigated, and therapies targeting EFNA1‐related pathways are well worth developing.

Furthermore, our studies identified cg12101586 (*CYP1A1*) to be significantly associated with increased risk of prostate cancer and breast cancer, suggesting the smoking‐related methylation at cg12101586 potentially affected the expression of *CYP1A1*, thus consequently increased the risk of cancers at both sites of prostate and breast. The CYP1A1 protein is one of the members of cytochrome P450 subfamily A and participates widely in the metabolic activation of carcinogens as a catalyst, which also situates it under the searchlight of potential carcinogenic effect.^43^ A cross‐sectional study including 542 healthy women form TwinsUK cohort reported a higher level of expression as well as hypomethylation of CYP1A1 in the current smokers than nonsmokers, supportively showing that the expression of CYP1A1 might be associated with smoking via methylation modification.^44^ Moreover, another valuable finding was provided by the same study that high methylation level of CYP1A1 could revert back after quitting smoking (especially 1 year after cessation with >50% reversal rate), suggesting that smoking cessation could be an effective strategy to alleviate the progression of smoking‐induced methylation at CYP1A1. The effect of CYP1A1 on the same cancers has been previously observed^45^ reporting that CYP1A1‐related pathway could exert the effect of driving cancer pathogenesis, progression and metastasis under the methylation related to smoking behaviors. Therefore, advocacy of smoking cessation needs to be addressed as an essential part of public health strategies combined with clinical treatment to provide early interventions.

The second MR analysis and overlaps of tissue‐specific eQTLs additionally indicated cg14142171 (*HLA‐L*) to have causal effect on cancers at breast and cervix through methylation modification. HLA is a highly polymorphic supergene which encodes the major histocompatibility complex (MHC) proteins in humans which was divided into three subregions: the HLA class I, II and III regions, in which HLA‐L belongs to class I as a pseudogene. HLA is suggested to be an underlying tumor suppressor, and was found with recurring mutations among varieties of malignancies.^46^ Studies have already dived into investigating the role of HLA gene variants in the pattern of cancers, suggesting the cooperative effect of multiple HLA regions of class I and II on the onset of cancers with infectious etiology or hematopoietic origin.^47^ Given that limited evidence was observed with HLA‐L and cancers, practices based on genetics or population yielding novel perspectives of association between HLA‐L gene and cancers need further documenting.

Moreover, cg07932199 (*ATXN2*) was remarkably indicated to have potentially same driving variants as those contributing to susceptibility to cancers at breast, colorectum, endometrium and lung, in agreement with previous findings. A study using sequencing analysis of exome and mRNA‐seq observed recurrent mutations of *ATXN2* in nonsmokers patients with lung cancer, revealing the nonnegligible role of *ATXN2* in the progress and prognosis of cancer patients.^48^ Similarly, a study analyzing the role of m^7^G‐lncRNAs using TCGA identified *ATXN2* as a key target regulated by m7G‐lncRNAs, with a higher expression in CRC.^49^ Since evidence on the association of *ATXN2* and multiple cancers is relatively sparse, our study provides supportive evidence from an epigenetic perspective of methylation, which requires deeper research in the future targeting related pathways.

Notably, a previous study investigating the causal effect of mQTLs at lung cancer‐related CpG sites with lung cancer suggested no confounding effects of smoking behaviors in the associations,^50^ which potentially indicated that there was possibly little overlap between smoking‐associated and lung cancer‐associated methylation pathways, and therefore might not cause confounding to each other. This finding also highlighted the importance of future investigations on the interaction and overlap of methylation among different trait is needed.

Our study has several strengths. First, we explore the association between smoking and 15 site‐specific cancers to provide a comprehensive perspective of the varying risks among different cancers responding to smoking, and to further investigate the cross‐cancer effect of smoking. Also, genetic instruments for smoking behaviors and multiple cancers were derived from the newest and largest GWASs to ensure accuracy and reliability. MR analyses were applied in our study to avoid reverse causality and to reduce the interference of confounding factors, and evidence was provided from both genetic and epigenetic perspectives by respectively utilizing SNPs and mQTLs as IVs, further highlighting the underlying role of methylation modification in carcinogenesis. Our study also has some limitations. We obtained methylation data from peripheral blood samples which could show a different methylation pattern from specific tissues,^51^ and our methylation data did not include information on time‐varying methylation changes. Nonetheless, we validated our main findings (eg, EFNA1, CYP1A1, HLA‐L, etc) further with colocalization and tissue‐specific expression evidence to enhance the reliability of the results as causal effects. The power of the analyses with some cancers (eg, biliary cancer, testis cancer, etc) might be attenuated due to their small case numbers. Horizontal pleiotropy is an inevitable problem when utilizing genetic variables, especially for phenotypes predicted by a few SNPs. However, we conducted sensitivity analyses, for example, MR Egger and MR‐PRESSO which could correct and remove outliers to ensure the robustness. Also, all GWASs were derived from cohort or studies of European ancestry, which impose restrictions on the extrapolation of our conclusion.

## 5 CONCLUSIONS

Our study found smoking behaviors to be genetically associated with multiple cancers, and provided further epigenetic perspective that DNA methylation at CpG sites could potentially act as a crucial part of carcinogenesis. Aberrant DNA methylation at several CpG sites related to smoking, including cg06639488 (*EFNA1*), cg12101586 (*CYP1A1*), cg14142171 (*HLA‐L*) and cg07932199 (*ATXN2*), were indicated with cross‐cancer carcinogenic effects.

## AUTHOR CONTRIBUTIONS

The work reported in the paper has been performed by the authors, unless clearly specified in the text. *Study conceptualization and design*: Xue Li, Evropi Theodoratou and Xiao Qian; *Data collection and curation*: Yajing Zhou, Xuan Zhou, Maria Timofeeva, Athina Spiliopoulou, Evropi Theodoratou and Xue Li; *Methodology establishment*: Xuan Zhou, Yajing Zhou and Xue Li; *Formal analysis and software support*: Yajing Zhou, Xuan Zhou and Xue Li. The original draft was written by Yajing Zhou; and the manuscript was revised and edited by Xuan Zhou, Xiao Qian, Xue Li and Evropi Theodoratou. All co‐authors had the opportunity to comment on the analysis and interpretation of the findings and approved the final version for publication.

## FUNDING INFORMATION

Xue Li is supported by the Natural Science Fund for Distinguished Young Scholars of Zhejiang Province (LR22H260001) and the National Nature Science Foundation of China (82204019). Yazhou He is supported by the NSFC (82103918) and Sichuan Provincial Nature Science Foundation (2022NSFSC1314). Evropi Theodoratou is supported by a CRUK Career Development Fellowship (C31250/A22804). Kefeng Ding is supported by the project of the regional diagnosis and treatment center of the Health Planning Committee (No. JBZX‐201903). This work is also funded by a grant to Malcolm G Dunlop as Project Leader with the MRC Human Genetics Unit Centre Grant (U127527198).

## CONFLICT OF INTEREST STATEMENT

The authors declare no conflicts of interest.

## ETHICS STATEMENT

All studies were approved by the irrespective institutional review boards and conducted with appropriate ethical criteria in each country and in accordance with the Declaration of Helsinki.

## Supporting information


**Data S1.** Supporting Information.

## Data Availability

The analysis results of our study are included in this published article and its supplementary information files. The UK Biobank and FinnGen dataset can be accessed through their access application process. Web resources: Genetics of DNA Methylation Consortium (GoDMC), http://mqtldb.godmc.org.uk; The Breast Cancer Association Consortium (BCAC), http://bcac.ccge.medschl.cam.ac.uk/; The Ovarian Cancer Association Consortium (OCAC), https://ocac.ccge.medschl.cam.ac.uk/; The International Lung Cancer Consortium (ILCCO), https://ilcco.iarc.fr/; Finngen Consortium, https://www.finngen.fi/en; UK Biobank, https://www.ukbiobank.ac.uk/. The data from the Genotype‐Tissue Expression (GTEx) Project 353 Portal were obtained on 08/18/2022. Further details and other data that support the findings of our study are available from the corresponding authors upon request.
