# Supplementary material for: Exploring the cross‐cancer effect of smoking and its fingerprints in blood DNA methylation on multiple cancers: A Mendelian randomization study
Source: Int J Cancer. 2023 Jul 14;153(8):1477–86. doi: 10.1002/ijc.34656 (PMC10952911; doi:10.1002/ijc.34656)
Supplement: Supplementary file 1 — Data S1. Supporting Information. [file IJC-153-1477-s001.pdf]

Exploring the cross-cancer effect of smoking and its fingerprints in blood DNA methylation on multiple cancers: a mendelian randomization study

Yajing Zhou†, Xuan Zhou†, Jing Sun, Lijuan Wang, Jianhui Zhao, Jie Chen, Shuai Yuan, Yazhou He, Maria Timofeeva, Athina Spiliopoulou, Ines Mesa-Eguiagaray, Susan M Farrington, Kefeng Ding, Malcolm Dunlop, Xiao Qian#*, Evropi Theodoratou#, Xue Li#*

Content

Supplementary tables **2**

Table S1. Detailed Information of exposure and outcome in two Mendelian randomization (MR) analyses. 2

Table S2. Genetic instruments for smoking behaviors. 4

Table S3. Result of the first MR analysis of smoking behaviors and cancers (using SNPs as genetic instrumental variables). 22

Table S4. Details of significant associations (FDR<0.05) between smoking-related aberrant CPG sites and cancers. 39

Table S5. MQTLs of CpG sites overlapped with expression. 44

Table S6. Colocalization analysis of CpG sites significantly associated with multiple cancers. 45

Supplementary figures **46**

Figure S1. Heatmap of CpG sites with cross-cancer effect. 46

Figure S2. Colocalization evidence for methylation at CpG site cg04521616 and susceptibility to breast cancer. 47

Figure S3. Colocalization evidence for methylation at CpG site cg06639488 and susceptibility to breast cancer. 48

Figure S4. Colocalization evidence for methylation at CpG site cg10666909 and susceptibility to breast cancer. 49

Figure S5. Colocalization evidence for methylation at CpG site cg20366110 and susceptibility to breast cancer. 50

Figure S6. Colocalization evidence for methylation at CpG site cg25839482 and susceptibility to breast cancer. 51

Figure S7. Colocalization evidence for methylation at CpG site cg03531211 and susceptibility to endometrial cancer. 52

Figure S8. Colocalization evidence for methylation at CpG site cg27424326 and susceptibility to liver cancer. 53

Figure S9. Colocalization evidence for methylation at CpG site cg20069688 and susceptibility to lung cancer. 54

Supplementary tables

Table S1. Detailed Information of exposure and outcome in two Mendelian randomization (MR) analyses.

Exposure/Outcome		Category	Numbers of IVs	GWAS/EWAS sources	Notes
Exposure of the first MR	Smoking behaviors	Age of smoking initiation	10	Liu M et al GWAS	SNPs were used as genetic proxies
		Smoking initiation	378		
		Smoking cessation	24		
		Lifetime smoking index (LSI)	126	Wootton et al GWAS	
Exposure of the second MR	Smoking-related DNA methylation	-	923,697	Joehanes R et al EWAS	mQTLs were used as genetic proxies of diffirentially methylated CpG sites
Outcome	Site-specific cancers	Biliary cancer	-	Finngen Consortium	SNPs were used as genetic proxies
		Brain cancer	-	Finngen Consortium	
		Breast cancer	65	BCAC	
		Cervix cancer	-	The UKB and GERA	
		Colorectal cancer	1,593	Li X et al GWAS	
		Endometrial cancer	9	O'Mara TA et al GWAS	
		Leukaemia	-	Finngen Consortium	
		Liver cancer	-	Finngen Consortium	
		Lung cancer	50	ILCCO	
		Ovarian cancer	12	OCAC	

Pancreatic cancer	-	The UKB and GERA
Prostate cancer	63	PRACTICAL Consortium
Rectum cancer	-	The UKB and GERA
Testis cancer	-	Finngen Consortium
multiple myeloma and malignant plasma cell neoplasms	-	Finngen Consortium

Abbreviations: IVs, instrumental variables; GWAS, genome-wide association study; EWAS, epigenome-wide association study; BCAC, the Breast Cancer Association Consortium; UKB, UK biobank; ILCCO, the International Lung Cancer Consortium; OCAC, the Ovarian Cancer Association Consortium; SNP, single nucleotide polymorphism; mQTLs, methylation quantitative trait loci.

Table S2. Genetic instruments for smoking behaviors.

Phenotype	SNP	Chr	Position	Effect Allele	Other Allele	EAF	Beta	SE	P value	F-statistics
Age of smoking initiation	rs7559982	2	63622309	A	T	0.564	-0.0172	0.0024	1.67E-12	49.8
	rs72853300	2	145638766	T	C	0.153	0.0190	0.0034	1.75E-08	31.8
	rs11915747	3	85699040	G	C	0.354	0.0202	0.0025	1.57E-15	63.5
	rs624833	4	2881256	G	T	0.302	0.0157	0.0026	2.36E-09	35.7
	rs2471711	4	28589079	T	C	0.152	-0.0192	0.0034	1.19E-08	32.5
	rs7682598	4	68000888	G	A	0.771	0.0173	0.0029	2.09E-09	35.9
	rs13136239	4	140908755	A	G	0.342	0.0148	0.0026	6.29E-09	33.7
	rs1403174	7	2032865	T	A	0.579	0.0155	0.0025	2.50E-10	40.0
	rs11780471	8	27344719	A	G	0.060	0.0330	0.0051	9.44E-11	41.9
Smoking initiation	rs12130857	1	7791461	A	G	0.325	-0.0180	0.0027	3.65E-11	43.8
	rs301807	1	8484823	G	A	0.570	0.0180	0.0026	2.50E-12	49.0
	rs3820277	1	18436657	T	G	0.526	-0.0188	0.0026	1.57E-13	54.5
	rs1889571	1	32195819	G	T	0.131	0.0222	0.0038	4.19E-09	34.5
	rs10914684	1	33795572	A	G	0.324	-0.0158	0.0027	6.32E-09	33.7
	rs2637869	1	38757237	A	G	0.297	0.0182	0.0028	6.54E-11	42.7
	rs12755632	1	41776623	G	A	0.316	-0.0154	0.0027	1.93E-08	31.6
	rs951740	1	44011737	A	G	0.625	0.0295	0.0026	3.82E-29	126.0
	rs925524	1	46496709	G	A	0.710	0.0156	0.0028	2.94E-08	30.7
	rs12022778	1	50603995	C	A	0.202	0.0268	0.0032	3.18E-17	71.2
	rs4912332	1	58815243	T	C	0.491	0.0141	0.0025	2.94E-08	30.7
	rs1937443	1	66469643	G	C	0.563	0.0204	0.0026	1.79E-15	63.3
	rs12740789	1	72752073	A	G	0.178	-0.0285	0.0033	1.18E-17	73.2
	rs10789369	1	73824909	G	A	0.615	-0.0234	0.0026	3.39E-19	80.2
	rs1514176	1	74991596	A	G	0.580	-0.0193	0.0026	7.67E-14	55.9

rs10873871	1	76689019	G	A	0.207	0.0175	0.0031	2.82E-08	30.8
rs11162019	1	87913176	T	C	0.363	-0.0155	0.0026	5.06E-09	34.2
rs1008078	1	91189731	T	C	0.402	0.0228	0.0026	1.63E-18	77.1
rs12027999	1	154206358	C	T	0.120	-0.0244	0.0039	5.33E-10	38.6
rs45444697	1	155034632	G	C	0.212	0.0197	0.0031	2.72E-10	39.9
rs2901785	1	174104743	A	G	0.446	-0.0173	0.0026	1.47E-11	45.6
rs147052174	1	179783167	T	G	0.017	0.0623	0.0098	2.30E-10	40.2
rs35656245	1	190957480	A	G	0.276	0.0159	0.0029	2.23E-08	31.3
rs12739243	1	210302043	C	T	0.221	-0.0213	0.0031	4.45E-12	47.9
rs12563365	1	236872829	A	G	0.556	0.0166	0.0026	1.05E-10	41.7
rs876793	1	237852083	C	T	0.349	-0.0179	0.0027	5.69E-11	42.9
rs114976176	2	264621	C	A	0.352	-0.0155	0.0027	6.04E-09	33.8
rs62106258	2	417167	C	T	0.047	-0.0455	0.0060	3.33E-14	57.5
rs6731872	2	624205	G	T	0.826	0.0316	0.0034	5.35E-21	88.4
rs1022376	2	22067213	C	T	0.516	-0.0147	0.0026	1.66E-08	31.9
rs61533748	2	22582968	C	T	0.384	0.0174	0.0026	2.82E-11	44.3
rs72790288	2	29513404	A	G	0.028	-0.0455	0.0077	3.28E-09	35.0
rs2710634	2	32808804	C	T	0.521	-0.0178	0.0026	3.36E-12	48.5
rs62137126	2	44250149	G	A	0.121	-0.0237	0.0039	1.31E-09	36.8
rs1004787	2	45159091	A	G	0.552	0.0284	0.0026	1.11E-28	123.0
rs7598402	2	50735943	G	C	0.492	-0.0147	0.0025	7.38E-09	33.4
rs10490159	2	51341259	T	C	0.394	0.0172	0.0026	3.86E-11	43.7
rs1518393	2	58171220	C	A	0.619	0.0169	0.0026	1.30E-10	41.3
rs17616642	2	59022210	G	A	0.247	-0.0166	0.0030	2.10E-08	31.4
rs2539706	2	59819545	A	G	0.530	0.0162	0.0026	1.95E-10	40.5
rs1863161	2	60139524	A	G	0.561	0.0153	0.0026	2.34E-09	35.7
rs359247	2	60477052	T	A	0.639	0.0220	0.0027	9.89E-17	69.0

rs62180324	2	63416606	A	G	0.212	-0.0195	0.0031	3.91E-10	39.2
rs6750107	2	80748807	A	G	0.387	0.0146	0.0026	2.60E-08	31.0
rs12714017	2	80999398	C	T	0.511	0.0154	0.0026	3.65E-09	34.8
rs56208390	2	83247997	G	A	0.123	0.0216	0.0039	2.68E-08	30.9
rs11692435	2	98275354	A	G	0.085	0.0251	0.0046	4.47E-08	29.9
rs13392222	2	100672408	C	A	0.139	-0.0234	0.0037	1.93E-10	40.5
rs1901477	2	104126983	G	A	0.511	0.0304	0.0026	2.07E-31	136.0
rs3811038	2	113240183	C	T	0.279	0.0191	0.0028	1.58E-11	45.4
rs34399632	2	137571174	G	A	0.232	0.0194	0.0030	1.46E-10	41.1
rs6756212	2	146140132	T	C	0.535	-0.0339	0.0026	3.49E-40	176.0
rs16826827	2	147825689	C	T	0.124	-0.0222	0.0039	9.17E-09	33.0
rs1445649	2	155682556	C	T	0.538	0.0206	0.0026	8.48E-16	64.8
rs12474587	2	162802993	T	G	0.429	0.0242	0.0026	4.83E-21	88.6
rs357304	2	164862639	C	T	0.727	0.0167	0.0029	5.40E-09	34.0
rs13007361	2	166250244	A	G	0.208	0.0175	0.0031	2.29E-08	31.2
rs7600835	2	172521827	A	G	0.342	-0.0151	0.0027	1.80E-08	31.7
rs6750529	2	182027603	T	C	0.744	0.0199	0.0029	9.26E-12	46.5
rs17229285	2	199523122	T	C	0.505	-0.0155	0.0025	1.27E-09	36.9
rs3115418	2	200936399	C	T	0.454	-0.0142	0.0026	2.79E-08	30.9
rs62193862	2	202843875	A	G	0.100	0.0238	0.0042	1.99E-08	31.5
rs4674916	2	225365635	A	C	0.328	-0.0180	0.0027	3.06E-11	44.1
rs4674993	2	226332033	G	A	0.200	-0.0240	0.0032	4.85E-14	56.8
rs11713899	3	2365026	C	A	0.171	0.0187	0.0034	3.15E-08	30.6
rs748832	3	16851202	G	A	0.371	0.0172	0.0026	6.60E-11	42.6
rs10446419	3	25725501	G	A	0.207	-0.0196	0.0031	5.05E-10	38.7
rs3172494	3	48731487	T	G	0.115	-0.0291	0.0040	3.40E-13	53.0
rs2526390	3	50192760	T	C	0.334	0.0205	0.0027	3.62E-14	57.4

rs2276825	3	52886605	C	T	0.245	0.0189	0.0030	1.89E-10	40.6
rs2306866	3	53766212	T	A	0.614	-0.0167	0.0026	1.89E-10	40.6
rs73831818	3	55988394	G	A	0.057	0.0320	0.0055	5.46E-09	34.0
rs7640107	3	59966156	T	C	0.431	-0.0142	0.0026	3.46E-08	30.4
rs2734390	3	60459291	G	A	0.372	0.0148	0.0026	2.09E-08	31.4
rs221988	3	64234307	C	A	0.384	-0.0149	0.0026	1.43E-08	32.2
rs11128203	3	71064431	A	T	0.530	0.0204	0.0026	1.29E-15	63.9
rs62246017	3	71483084	A	G	0.323	-0.0162	0.0027	3.03E-09	35.2
rs4543050	3	74954560	T	A	0.816	0.0222	0.0033	1.45E-11	45.6
rs6782116	3	77176032	T	C	0.415	-0.0147	0.0026	1.46E-08	32.1
rs13066050	3	81325861	T	C	0.208	0.0188	0.0031	1.93E-09	36.0
rs12633090	3	83241365	C	G	0.182	-0.0230	0.0033	3.16E-12	48.6
rs1549979	3	85460131	T	C	0.615	-0.0245	0.0026	8.80E-21	87.4
rs6437769	3	107997514	T	C	0.581	0.0142	0.0026	3.74E-08	30.3
rs9288999	3	114147927	A	G	0.735	0.0174	0.0029	1.50E-09	36.5
rs6438436	3	117822149	T	C	0.816	0.0247	0.0033	5.33E-14	56.6
rs9826984	3	131945722	A	G	0.542	-0.0141	0.0026	3.87E-08	30.2
rs2279829	3	147106319	T	C	0.216	-0.0174	0.0031	2.05E-08	31.5
rs2319545	3	147719648	A	C	0.149	0.0232	0.0036	8.30E-11	42.2
rs10935779	3	149543102	T	C	0.415	-0.0143	0.0026	2.95E-08	30.7
rs1714521	3	158284861	C	A	0.411	-0.0163	0.0026	3.07E-10	39.6
rs1449012	3	159048333	T	C	0.463	-0.0154	0.0026	1.77E-09	36.2
rs9850597	3	161761866	A	G	0.816	-0.0186	0.0033	1.65E-08	31.9
rs1187820	3	173072584	T	C	0.439	-0.0143	0.0026	2.69E-08	30.9
rs16828799	3	173353739	T	G	0.156	0.0198	0.0035	1.83E-08	31.7
rs9841807	3	175718927	T	C	0.273	0.0163	0.0029	1.35E-08	32.3
rs7631379	3	181409057	C	T	0.206	0.0208	0.0032	3.94E-11	43.6

rs4140932	4	15458598	A	T	0.431	-0.0140	0.0026	4.89E-08	29.8
rs59537158	4	28246049	T	C	0.214	0.0225	0.0031	4.62E-13	52.4
rs55944129	4	29082156	C	T	0.267	-0.0176	0.0029	1.06E-09	37.2
rs58400863	4	31184484	A	G	0.347	-0.0202	0.0027	4.89E-14	56.8
rs7657022	4	35501032	G	A	0.489	0.0183	0.0025	7.34E-13	51.5
rs112725451	4	68017710	T	C	0.169	0.0261	0.0034	1.65E-14	58.9
rs1160685	4	94052854	G	C	0.450	0.0153	0.0026	2.31E-09	35.7
rs1435479	4	94550450	T	G	0.287	0.0164	0.0028	5.68E-09	33.9
rs3934797	4	112467612	A	G	0.182	-0.0213	0.0033	1.12E-10	41.6
rs71602617	4	136406155	T	C	0.216	-0.0178	0.0032	2.10E-08	31.4
rs7696257	4	137474783	A	G	0.366	0.0153	0.0026	6.78E-09	33.6
rs13109980	4	140886963	A	G	0.326	-0.0222	0.0027	3.37E-16	66.6
rs1116690	4	143510148	G	A	0.742	0.0163	0.0029	2.16E-08	31.3
rs13110073	4	147797913	C	T	0.395	-0.0246	0.0026	3.24E-21	89.4
rs62340589	4	176875795	C	G	0.201	0.0174	0.0032	4.31E-08	30.0
rs12517438	5	30842054	G	T	0.538	0.0154	0.0026	1.89E-09	36.1
rs35375873	5	43190647	C	G	0.110	-0.0270	0.0041	3.29E-11	44.0
rs986714	5	50821338	T	A	0.445	-0.0160	0.0026	4.13E-10	39.1
rs71592686	5	60121271	C	T	0.274	0.0207	0.0029	3.85E-13	52.7
rs2028269	5	79308315	A	G	0.399	0.0162	0.0026	5.19E-10	38.6
rs6874731	5	80263865	G	T	0.484	0.0153	0.0025	1.83E-09	36.1
rs6452785	5	87685500	T	C	0.474	-0.0269	0.0026	4.69E-26	111.0
rs10805858	5	88873832	T	A	0.335	0.0181	0.0027	1.88E-11	45.1
rs42417	5	94198290	T	C	0.691	0.0169	0.0028	8.27E-10	37.7
rs72780746	5	103929588	C	T	0.173	-0.0258	0.0034	2.05E-14	58.5
rs10060196	5	106455988	A	C	0.581	0.0183	0.0026	1.29E-12	50.3
rs72789626	5	106825618	A	T	0.136	-0.0256	0.0037	5.13E-12	47.6

rs17165769	5	107365642	G	A	0.395	0.0159	0.0026	9.56E-10	37.4
rs329124	5	133865452	G	A	0.428	-0.0164	0.0026	1.96E-10	40.5
rs1385108	5	154839646	T	C	0.239	0.0187	0.0030	3.84E-10	39.2
rs1173461	5	157707571	T	C	0.327	0.0166	0.0027	9.51E-10	37.4
rs11956866	5	161018271	G	T	0.567	-0.0148	0.0026	7.82E-09	33.3
rs3909281	5	165096435	G	T	0.536	0.0211	0.0026	1.62E-16	68.0
rs3843905	5	165427280	T	C	0.403	-0.0151	0.0026	5.41E-09	34.0
rs6890961	5	166778503	T	C	0.624	-0.0193	0.0026	2.13E-13	53.9
rs4044321	5	166989513	G	A	0.644	-0.0226	0.0027	1.75E-17	72.4
rs2173019	5	167614971	A	T	0.177	0.0282	0.0033	2.98E-17	71.4
rs10042827	5	170299916	C	T	0.681	0.0167	0.0027	9.41E-10	37.4
rs359431	5	173288534	T	C	0.560	-0.0142	0.0026	3.16E-08	30.6
rs1150668	6	28129789	G	T	0.419	-0.0185	0.0026	8.54E-13	51.2
rs1632941	6	29796685	C	T	0.460	-0.0158	0.0026	6.67E-10	38.1
rs3218116	6	41901763	T	C	0.256	-0.0198	0.0029	1.05E-11	46.2
rs160631	6	52895230	G	T	0.731	-0.0173	0.0029	1.87E-09	36.1
rs7743165	6	67521222	G	T	0.495	0.0193	0.0025	4.15E-14	57.1
rs10945141	6	69470709	A	G	0.263	0.0181	0.0029	3.59E-10	39.3
rs17554906	6	92226609	C	G	0.444	0.0142	0.0026	3.14E-08	30.6
rs619087	6	94175279	G	A	0.422	0.0143	0.0026	3.10E-08	30.6
rs6568832	6	97702876	A	G	0.754	0.0189	0.0030	1.74E-10	40.7
rs12195240	6	98636905	A	G	0.285	0.0249	0.0028	1.08E-18	77.9
rs6936160	6	100347745	T	C	0.698	0.0201	0.0028	4.20E-13	52.5
rs3800227	6	108994161	G	A	0.742	0.0172	0.0029	3.64E-09	34.8
rs118202	6	111658371	T	G	0.812	-0.0367	0.0033	1.90E-29	127.0
rs73008357	6	156431856	C	A	0.121	-0.0223	0.0040	2.44E-08	31.1
rs9331343	6	157738258	C	T	0.568	-0.0141	0.0026	3.90E-08	30.2

rs1737329	6	163807748	G	C	0.742	0.0170	0.0029	5.08E-09	34.2
rs6948707	7	1870794	G	T	0.419	0.0243	0.0026	4.24E-21	88.9
rs13237637	7	3503207	C	G	0.485	-0.0237	0.0025	1.54E-20	86.3
rs7809303	7	69484366	A	G	0.325	-0.0214	0.0027	3.48E-15	62.0
rs7802996	7	77771983	T	C	0.166	-0.0209	0.0034	1.06E-09	37.2
rs1030015	7	78139581	T	G	0.520	0.0143	0.0026	2.15E-08	31.4
rs4727189	7	88442568	C	T	0.344	0.0149	0.0027	3.00E-08	30.7
rs76841737	7	91281409	G	C	0.103	-0.0231	0.0042	3.26E-08	30.5
rs11768481	7	96629103	A	C	0.340	-0.0186	0.0027	5.23E-12	47.6
rs1799068	7	97707069	T	G	0.379	0.0166	0.0026	2.59E-10	40.0
rs13437771	7	99071478	G	A	0.155	-0.0271	0.0035	1.39E-14	59.3
rs11766326	7	111100585	C	T	0.506	-0.0175	0.0026	1.79E-11	45.2
rs6968380	7	114940159	A	G	0.681	-0.0234	0.0027	1.05E-17	73.4
rs10233018	7	117523709	G	A	0.516	0.0246	0.0025	4.77E-22	93.2
rs10953957	7	121954709	A	G	0.386	0.0144	0.0026	3.66E-08	30.3
rs77283305	7	132593831	A	G	0.306	-0.0152	0.0028	3.91E-08	30.2
rs10279261	7	133589846	A	G	0.618	-0.0189	0.0026	6.05E-13	51.8
rs4326350	8	10763655	G	C	0.493	-0.0176	0.0026	5.16E-12	47.6
rs11783093	8	27425349	T	C	0.158	-0.0471	0.0035	2.07E-41	182.0
rs7836565	8	52569449	T	C	0.718	-0.0155	0.0028	4.36E-08	30.0
rs13261666	8	59814666	T	G	0.517	-0.0200	0.0025	4.36E-15	61.5
rs3850736	8	64912021	G	C	0.474	0.0191	0.0026	6.43E-14	56.2
rs2063976	8	91096366	T	C	0.665	-0.0202	0.0027	7.45E-14	55.9
rs6986430	8	93048104	C	T	0.222	-0.0243	0.0031	1.99E-15	63.1
rs9987376	8	93190014	G	T	0.574	-0.0205	0.0026	2.01E-15	63.1
rs290601	8	115374642	T	C	0.274	0.0163	0.0029	1.14E-08	32.6
rs3847244	9	3025368	T	C	0.470	0.0187	0.0026	2.60E-13	53.5

rs11791671	9	3398679	T	C	0.067	0.0279	0.0051	4.24E-08	30.0
rs7024924	9	8282399	C	T	0.174	0.0189	0.0034	1.90E-08	31.6
rs1931431	9	11161799	C	G	0.478	0.0182	0.0026	8.56E-13	51.1
rs7867822	9	20676454	G	A	0.673	-0.0151	0.0027	2.76E-08	30.9
rs10966092	9	23831658	C	T	0.267	-0.0205	0.0029	1.12E-12	50.6
rs10969352	9	29747488	A	T	0.500	0.0143	0.0025	1.82E-08	31.7
rs4877285	9	81354129	A	G	0.668	-0.0181	0.0027	2.10E-11	44.9
rs1930371	9	81444104	T	C	0.241	-0.0172	0.0030	7.09E-09	33.5
rs2378662	9	86707289	A	G	0.541	0.0152	0.0026	2.67E-09	35.4
rs1927901	9	120519111	C	T	0.553	-0.0142	0.0026	3.10E-08	30.6
rs4837631	9	122061948	T	C	0.446	-0.0154	0.0026	2.03E-09	35.9
rs1759433	9	128073097	A	G	0.480	0.0154	0.0026	1.69E-09	36.3
rs34553878	9	134334588	G	A	0.111	0.0247	0.0041	1.17E-09	37.0
rs7026534	9	134907263	G	T	0.704	-0.0166	0.0028	2.68E-09	35.4
rs10858334	9	137989785	G	C	0.140	0.0229	0.0038	1.18E-09	37.0
rs10905461	10	8803551	C	T	0.748	-0.0164	0.0029	2.36E-08	31.2
rs7920501	10	10043159	A	T	0.465	-0.0155	0.0026	1.25E-09	36.9
rs1291821	10	11133823	G	A	0.534	0.0145	0.0026	1.39E-08	32.2
rs11258417	10	13533053	T	C	0.391	-0.0145	0.0026	2.71E-08	30.9
rs7072776	10	22032942	G	A	0.712	-0.0220	0.0028	5.66E-15	61.0
rs2796793	10	36634124	A	G	0.452	0.0145	0.0026	1.55E-08	32.0
rs1733760	10	56698174	C	T	0.510	0.0148	0.0025	6.70E-09	33.6
rs7921378	10	63674885	C	G	0.482	-0.0233	0.0025	6.10E-20	83.6
rs11594623	10	103960351	C	T	0.234	0.0274	0.0030	7.45E-20	83.2
rs28408682	10	104403310	G	A	0.600	0.0167	0.0026	1.41E-10	41.1
rs12244388	10	104640052	A	G	0.350	0.0258	0.0027	4.31E-22	93.4
rs11192347	10	106929313	A	G	0.104	-0.0265	0.0043	6.15E-10	38.3

rs10885480	10	115378364	C	T	0.284	-0.0187	0.0028	3.83E-11	43.7
rs4752018	10	118678712	A	C	0.231	0.0189	0.0030	4.42E-10	38.9
rs9423279	10	125680419	G	C	0.645	-0.0186	0.0027	3.06E-12	48.7
rs6265	11	27679916	T	C	0.188	-0.0293	0.0033	2.81E-19	80.6
rs62618693	11	32956492	T	C	0.043	-0.0353	0.0063	2.09E-08	31.4
rs2939756	11	41436297	A	G	0.480	-0.0157	0.0026	7.45E-10	37.9
rs1381775	11	42442826	C	T	0.712	-0.0156	0.0028	2.79E-08	30.8
rs2959084	11	46078656	A	G	0.705	0.0171	0.0028	9.82E-10	37.4
rs3740977	11	46393574	C	T	0.167	0.0195	0.0034	1.17E-08	32.5
rs61886926	11	64133552	T	C	0.384	-0.0179	0.0026	7.30E-12	46.9
rs61884449	11	64485193	T	C	0.149	0.0200	0.0036	2.32E-08	31.2
rs644740	11	65561468	T	C	0.457	-0.0141	0.0026	3.67E-08	30.3
rs7943721	11	73309393	A	G	0.829	-0.0212	0.0034	3.58E-10	39.3
rs7929518	11	85980958	G	A	0.773	0.0192	0.0030	2.55E-10	40.0
rs586699	11	92289734	A	G	0.543	-0.0148	0.0026	7.29E-09	33.5
rs76460663	11	111979741	G	C	0.041	-0.0423	0.0064	4.15E-11	43.5
rs2155646	11	112912811	C	T	0.400	0.0378	0.0026	9.44E-48	211.0
rs1713676	11	113660576	G	A	0.523	-0.0167	0.0026	5.38E-11	43.0
rs238896	11	113994505	A	G	0.490	-0.0169	0.0025	3.65E-11	43.8
rs540860	11	121530888	G	A	0.543	0.0176	0.0026	5.75E-12	47.4
rs1106363	11	131966264	T	C	0.345	0.0174	0.0027	9.20E-11	42.0
rs2010921	11	132098205	A	G	0.311	0.0174	0.0028	2.47E-10	40.1
rs11057005	12	16748721	G	A	0.441	-0.0157	0.0026	9.12E-10	37.5
rs13906	12	49952394	T	C	0.109	-0.0245	0.0041	1.98E-09	36.0
rs4759229	12	56474480	G	A	0.656	0.0156	0.0027	6.53E-09	33.7
rs7969559	12	69655167	G	A	0.713	-0.0170	0.0028	1.53E-09	36.5
rs7134009	12	75263193	C	T	0.287	-0.0158	0.0029	4.30E-08	30.0

rs77215829	12	112618346	C	A	0.131	-0.0240	0.0038	2.02E-10	40.4
rs1109480	12	121083279	A	G	0.384	-0.0167	0.0026	1.84E-10	40.6
rs11611651	12	133380790	A	G	0.087	0.0271	0.0045	2.05E-09	35.9
rs17197663	13	38172867	A	G	0.125	-0.0216	0.0039	2.06E-08	31.4
rs4264267	13	38359676	T	C	0.527	0.0148	0.0026	6.82E-09	33.6
rs61959481	13	55834929	A	G	0.210	-0.0203	0.0031	7.95E-11	42.3
rs9538162	13	59265043	C	T	0.416	0.0174	0.0026	1.76E-11	45.2
rs55786907	13	59871584	G	A	0.162	0.0194	0.0035	1.84E-08	31.7
rs4886207	13	60705792	C	T	0.637	-0.0162	0.0026	8.78E-10	37.6
rs9540731	13	66949370	T	C	0.509	-0.0177	0.0025	3.42E-12	48.4
rs9545155	13	80191873	C	T	0.478	-0.0161	0.0026	3.04E-10	39.7
rs1772572	13	81191176	A	C	0.324	-0.0169	0.0027	5.62E-10	38.4
rs75674569	13	96823724	A	G	0.100	-0.0253	0.0043	2.58E-09	35.5
rs7333559	13	100546450	A	G	0.783	-0.0232	0.0031	5.94E-14	56.4
rs1108130	13	100648356	A	T	0.212	0.0239	0.0031	1.57E-14	59.0
rs12855717	13	101252635	T	C	0.538	0.0155	0.0026	1.22E-09	36.9
rs12878369	14	28346502	A	C	0.415	0.0174	0.0026	1.60E-11	45.4
rs9323328	14	58653514	G	A	0.537	-0.0142	0.0026	2.55E-08	31.0
rs1811739	14	77529375	A	G	0.248	0.0183	0.0030	5.97E-10	38.3
rs8005334	14	79563654	G	T	0.360	0.0167	0.0027	3.44E-10	39.4
rs34940743	14	80102233	G	A	0.346	0.0159	0.0027	2.80E-09	35.3
rs2925128	14	98362355	T	C	0.385	0.0168	0.0027	3.67E-10	39.3
rs1381287	14	98597552	T	C	0.467	0.0180	0.0026	1.81E-12	49.7
rs55913542	14	99693843	T	G	0.175	0.0186	0.0034	3.25E-08	30.6
rs1435672	15	36399479	C	T	0.560	0.0141	0.0026	3.82E-08	30.2
rs281296	15	47685010	A	G	0.357	0.0247	0.0027	1.59E-20	86.2
rs56902655	15	63898709	G	T	0.136	-0.0219	0.0037	4.09E-09	34.6

rs2289791	15	67476952	T	G	0.247	-0.0177	0.0030	2.01E-09	36.0
rs60833441	15	74048768	G	A	0.461	-0.0143	0.0026	2.28E-08	31.2
rs62007780	15	78025464	T	G	0.416	-0.0159	0.0026	7.48E-10	37.9
rs4310804	15	96858409	G	C	0.247	-0.0182	0.0030	7.55E-10	37.9
rs8027457	15	99204101	C	T	0.511	0.0153	0.0025	1.88E-09	36.1
rs1139897	16	720986	A	G	0.230	-0.0241	0.0030	1.77E-15	63.3
rs11076962	16	5811367	C	T	0.279	0.0183	0.0028	1.20E-10	41.5
rs7192140	16	10173748	C	T	0.498	-0.0169	0.0025	3.40E-11	43.9
rs9922607	16	17570220	T	C	0.200	-0.0222	0.0032	3.42E-12	48.4
rs9941217	16	18050926	G	C	0.352	-0.0186	0.0027	3.50E-12	48.4
rs7188873	16	24727064	G	A	0.613	0.0203	0.0026	8.46E-15	60.2
rs6497840	16	25351633	A	G	0.707	0.0228	0.0029	2.01E-15	63.1
rs4785187	16	49766772	A	G	0.223	0.0200	0.0031	6.55E-11	42.6
rs8050598	16	49891964	T	C	0.254	0.0187	0.0029	1.76E-10	40.7
rs12918191	16	50945156	G	A	0.243	-0.0197	0.0030	3.14E-11	44.1
rs9302604	16	69576894	G	A	0.435	0.0187	0.0026	3.29E-13	53.0
rs62052916	16	72574550	T	A	0.070	-0.0319	0.0050	1.62E-10	40.9
rs4788676	16	72950468	C	T	0.229	-0.0177	0.0030	4.92E-09	34.2
rs117657830	16	75766873	G	A	0.042	-0.0378	0.0064	3.18E-09	35.1
rs1050847	16	87443734	T	C	0.559	-0.0148	0.0026	7.37E-09	33.4
rs11642231	16	89608702	A	G	0.369	-0.0156	0.0026	3.44E-09	34.9
rs4790874	17	1995177	T	C	0.532	0.0174	0.0026	8.43E-12	46.7
rs28441558	17	7803118	C	T	0.056	-0.0356	0.0055	1.24E-10	41.4
rs11651955	17	16235462	A	G	0.499	-0.0140	0.0025	3.74E-08	30.3
rs67777803	17	27323322	T	G	0.172	-0.0246	0.0034	3.18E-13	53.1
rs2344976	17	30685935	C	T	0.612	-0.0151	0.0026	7.98E-09	33.3
rs17692129	17	44793283	T	C	0.331	0.0196	0.0027	4.57E-13	52.4

rs75919030	17	50193197	C	T	0.267	-0.0210	0.0029	3.35E-13	53.0
rs2587507	17	77790135	C	T	0.502	-0.0147	0.0025	8.69E-09	33.1
rs34342129	18	5872472	C	T	0.509	-0.0143	0.0025	2.13E-08	31.4
rs4476253	18	25253297	A	G	0.240	-0.0185	0.0030	5.78E-10	38.4
rs7505855	18	31696075	T	C	0.586	-0.0170	0.0026	5.31E-11	43.1
rs8096225	18	36921851	C	A	0.703	0.0155	0.0028	2.63E-08	31.0
rs67050670	18	39297254	G	A	0.229	-0.0203	0.0030	2.34E-11	44.7
rs72898831	18	42658643	G	A	0.155	-0.0244	0.0035	4.14E-12	48.1
rs1373178	18	49967811	G	T	0.588	-0.0203	0.0026	4.16E-15	61.6
rs62098013	18	50863861	A	G	0.365	0.0177	0.0026	2.24E-11	44.8
rs72938304	18	53661743	A	G	0.113	-0.0272	0.0040	1.36E-11	45.7
rs11872397	18	72535282	A	G	0.253	-0.0171	0.0029	5.20E-09	34.1
rs71367544	18	77574374	T	C	0.203	0.0206	0.0032	8.54E-11	42.1
rs76608582	19	4474725	A	C	0.049	-0.0345	0.0059	4.88E-09	34.2
rs10853981	19	4965064	A	G	0.330	0.0148	0.0027	4.88E-08	29.8
rs113230003	19	18460956	A	G	0.255	-0.0189	0.0029	1.05E-10	41.7
rs8103660	19	18566395	C	T	0.354	0.0158	0.0027	3.03E-09	35.2
rs117734003	19	51129745	C	G	0.067	0.0303	0.0051	2.57E-09	35.5
rs1126757	19	55879872	T	C	0.473	0.0142	0.0026	2.92E-08	30.8
rs6050446	20	25195509	G	A	0.971	0.0544	0.0076	8.80E-13	51.1
rs6073075	20	42015801	A	T	0.824	-0.0187	0.0034	2.44E-08	31.1
rs910912	20	54462393	C	T	0.739	-0.0168	0.0029	7.82E-09	33.3
rs6011779	20	61984317	T	C	0.806	-0.0192	0.0032	2.83E-09	35.3
rs3810496	20	62406886	C	T	0.619	0.0159	0.0026	1.54E-09	36.5
rs4818005	21	40588819	A	G	0.581	-0.0204	0.0026	1.09E-14	59.7
rs4822102	22	42698430	T	C	0.618	-0.0165	0.0026	2.78E-10	39.8
rs9627272	22	46442288	C	G	0.407	-0.0155	0.0026	2.42E-09	35.6

Smoking cessation	rs112187834	2	23953454	A	T	0.140	0.0334	0.0056	2.81E-09	35.3
	rs7617480	3	49210732	C	A	0.773	-0.0329	0.0047	1.68E-12	49.8
	rs12203592	6	396321	T	C	0.176	-0.0292	0.0051	1.21E-08	32.5
	rs707968	6	35058117	G	A	0.681	0.0233	0.0042	2.76E-08	30.9
	rs7778443	7	32314690	C	T	0.618	-0.0230	0.0040	1.04E-08	32.8
	rs1565735	8	27426077	A	T	0.199	-0.0346	0.0049	1.54E-12	50.0
	rs60749569	8	42602668	T	A	0.080	-0.0401	0.0072	2.68E-08	30.9
	rs9409844	9	136461851	A	G	0.045	-0.0586	0.0094	4.37E-10	38.9
	rs3025327	9	136467344	C	G	0.107	0.0786	0.0063	1.19E-35	155.0
	rs1611124	9	136509275	T	G	0.068	-0.0453	0.0078	5.26E-09	34.1
	rs7109376	11	16372431	A	T	0.279	0.0281	0.0044	1.14E-10	41.6
	rs591143	15	47647755	T	C	0.592	-0.0243	0.0040	1.14E-09	37.1
	rs3866543	15	76629609	G	T	0.523	0.0222	0.0039	1.35E-08	32.3
	rs518425	15	78883813	G	A	0.285	-0.0305	0.0043	1.72E-12	49.8
	rs56113850	19	41353107	C	T	0.567	-0.0576	0.0039	1.61E-48	214.0
	rs6011779	20	61984317	T	C	0.806	-0.0500	0.0050	9.89E-24	101.0
	rs6089904	20	62018289	T	A	0.047	-0.0642	0.0093	4.01E-12	48.1
	rs9607805	22	41854446	T	C	0.725	0.0295	0.0044	1.37E-11	45.7
Lifetime smoking index	rs1193237	1	7526486	G	C	0.439	-0.011	0.002	2.80E-08	27.6
	rs4949465	1	32178489	T	C	0.870	-0.017	0.003	1.70E-08	30.2
	rs549845	1	44076469	G	A	0.301	0.016	0.002	8.30E-14	49.8
	rs1933270	1	49977965	T	G	0.364	0.013	0.002	1.50E-10	36.2
	rs7528604	1	66407352	G	A	0.566	0.014	0.002	5.70E-12	44.6
	rs11210229	1	73860028	A	G	0.384	0.017	0.002	2.00E-16	63.3
	rs7553348	1	75005067	G	A	0.438	0.014	0.002	5.20E-12	44.7
	rs10922907	1	91193049	A	T	0.451	0.015	0.002	3.00E-13	51.6
	rs1931263	1	96175101	G	T	0.510	-0.011	0.002	4.00E-08	28.0

rs7519626	1	99514554	C	T	0.324	0.012	0.002	1.20E-08	29.2
rs9435340	1	107593201	T	A	0.344	0.012	0.002	1.20E-08	30.1
rs10918701	1	162090536	G	A	0.372	0.012	0.002	2.10E-08	31.1
rs2867112	2	651349	T	G	0.835	0.021	0.003	4.80E-15	56.2
rs6741228	2	22548774	T	C	0.433	0.011	0.002	1.60E-08	27.5
rs62135536	2	44326028	C	T	0.968	0.035	0.006	8.00E-10	35.1
rs7569203	2	45154418	A	C	0.689	-0.016	0.002	7.40E-13	50.8
rs13016665	2	57995348	C	A	0.577	-0.012	0.002	1.80E-09	32.5
rs4671357	2	60136176	T	C	0.519	-0.014	0.002	1.10E-11	45.3
rs359243	2	60475509	T	C	0.393	-0.013	0.002	9.50E-10	37.3
rs2678670	2	104469564	A	T	0.486	0.013	0.002	3.10E-10	39.1
rs62155874	2	105973094	A	G	0.873	-0.024	0.003	5.20E-16	59.1
rs3811038	2	113240183	T	C	0.724	-0.014	0.002	8.90E-10	36.2
rs2890772	2	146175106	G	T	0.413	-0.020	0.002	2.10E-22	89.8
rs62175972	2	161362830	T	C	0.966	0.031	0.006	1.70E-08	29.2
rs3769949	2	166199284	T	A	0.528	-0.012	0.002	2.50E-09	33.2
rs13009008	2	174043233	A	G	0.328	0.012	0.002	4.60E-09	29.4
rs4473348	2	182073742	A	T	0.250	-0.015	0.002	6.40E-11	39.0
rs12623702	2	202885506	A	G	0.613	-0.014	0.002	7.70E-12	43.0
rs6779302	3	16859710	G	T	0.633	-0.013	0.002	1.20E-09	36.3
rs6778080	3	49317338	T	C	0.267	0.016	0.002	1.30E-12	46.4
rs775758	3	77582005	A	T	0.433	0.012	0.002	1.10E-08	32.7
rs421983	3	84892866	T	C	0.519	0.013	0.002	3.30E-10	39.0
rs326341	3	107811142	G	A	0.525	0.014	0.002	1.20E-11	45.2
rs73220544	3	131074511	A	C	0.842	-0.016	0.003	1.50E-08	31.5
rs9842947	3	157412246	C	T	0.326	-0.013	0.002	3.10E-09	34.4
rs624833	4	2881256	T	G	0.695	0.013	0.002	6.60E-10	33.2

rs61796681	4	23678196	A	T	0.912	-0.019	0.004	4.20E-08	26.8
rs317021	4	35418368	T	A	0.814	-0.017	0.003	1.10E-10	40.5
rs72678864	4	112422145	G	A	0.829	0.018	0.003	1.60E-11	42.5
rs17576594	4	147952241	G	A	0.724	0.016	0.002	1.70E-12	47.3
rs11948770	5	13246336	T	C	0.768	-0.015	0.002	4.90E-10	37.1
rs71627581	5	43161351	G	A	0.889	0.019	0.003	1.60E-09	33.0
rs10052591	5	50812738	T	C	0.573	0.012	0.002	2.10E-09	32.6
rs4571506	5	87756918	C	T	0.540	0.011	0.002	1.50E-08	27.8
rs4957528	5	106420589	A	C	0.208	-0.015	0.002	4.20E-09	34.3
rs329120	5	133861756	C	T	0.581	0.014	0.002	6.30E-12	44.2
rs986391	5	166993972	G	A	0.367	0.016	0.002	9.40E-15	55.0
rs13153393	5	167604213	A	G	0.884	-0.020	0.003	2.50E-10	38.0
rs245774	5	170530930	A	G	0.272	-0.013	0.002	7.40E-09	31.0
rs6935954	6	26255451	A	G	0.421	0.014	0.002	8.20E-12	44.2
rs2254710	6	37477000	C	A	0.236	0.013	0.002	3.50E-08	28.2
rs2894808	6	52861990	T	A	0.922	-0.022	0.004	3.50E-09	32.2
rs12202536	6	67475273	A	G	0.513	-0.012	0.002	2.80E-09	33.3
rs7766610	6	111707821	C	A	0.183	0.018	0.003	2.20E-12	44.8
rs1922018	7	3560401	C	T	0.364	0.014	0.002	3.00E-12	42.0
rs10226228	7	32315613	A	G	0.630	-0.016	0.002	2.00E-15	55.2
rs11768481	7	96629103	C	A	0.666	0.013	0.002	9.90E-10	34.8
rs6962772	7	99081730	A	G	0.846	0.016	0.003	7.80E-09	30.9
rs10282292	7	111092478	C	T	0.362	0.013	0.002	5.90E-10	36.1
rs2401924	7	115057862	G	C	0.502	0.015	0.002	2.70E-14	52.1
rs7807019	7	117543063	A	G	0.540	-0.015	0.002	6.70E-14	51.7
rs6957896	7	132309592	C	T	0.503	-0.011	0.002	4.50E-08	28.0
rs4731925	7	132664757	C	T	0.316	-0.012	0.002	2.60E-08	28.8

rs35169606	8	9604066	T	G	0.612	0.013	0.002	1.20E-09	37.1
rs11783093	8	27425349	C	T	0.839	0.023	0.003	1.20E-16	66.1
rs2062882	8	91839576	G	A	0.587	-0.012	0.002	1.10E-08	32.3
rs72674867	8	95578201	A	T	0.765	0.013	0.002	3.80E-08	28.1
rs4543592	9	3014254	T	C	0.520	-0.012	0.002	4.50E-10	33.3
rs7039819	9	82430418	G	A	0.427	0.013	0.002	5.10E-10	38.3
rs1246265	9	86761745	T	C	0.305	-0.013	0.002	4.20E-09	33.2
rs1221148	9	122046875	C	G	0.587	0.013	0.002	7.30E-11	37.9
rs13296519	9	128471924	G	T	0.606	-0.014	0.002	8.10E-12	43.3
rs113382419	9	136463019	C	A	0.889	-0.041	0.003	3.00E-37	153.6
rs11255908	10	8802912	T	G	0.743	-0.015	0.002	2.30E-10	39.8
rs2675638	10	63576286	G	A	0.581	0.012	0.002	1.30E-09	32.4
rs10823968	10	74738269	A	T	0.633	0.012	0.002	2.10E-08	31.0
rs17553262	10	92912773	A	C	0.885	-0.018	0.003	5.30E-09	30.5
rs7077678	10	104438565	C	T	0.623	0.012	0.002	2.60E-09	31.3
rs12244388	10	104640052	G	A	0.661	-0.019	0.002	1.40E-19	74.9
rs3896224	10	106467853	A	G	0.585	0.014	0.002	1.10E-11	44.0
rs34866095	11	16377356	A	G	0.686	-0.012	0.002	1.20E-08	28.7
rs75742406	11	17070365	G	A	0.739	0.014	0.002	1.30E-09	35.0
rs17309874	11	27667236	G	A	0.740	-0.016	0.002	9.70E-13	45.6
rs4391802	11	28674592	A	G	0.707	0.015	0.002	1.40E-11	43.1
rs112282219	11	46632809	G	A	0.959	-0.033	0.005	3.80E-11	39.6
rs9919670	11	112877304	G	A	0.612	-0.022	0.002	7.60E-27	106.4
rs74086911	12	50015942	G	A	0.925	0.021	0.004	2.10E-08	28.3
rs7297175	12	56473808	T	C	0.431	-0.012	0.002	6.60E-09	32.7
rs10879871	12	75380511	T	G	0.343	-0.014	0.002	5.00E-11	40.9
rs12831617	12	84758368	C	T	0.764	-0.013	0.002	1.90E-08	28.2

rs6562474	13	67332812	C	G	0.651	0.012	0.002	1.00E-08	30.3
rs7333559	13	100546450	G	A	0.212	0.015	0.002	3.20E-10	34.8
rs860326	14	57342912	C	T	0.428	0.012	0.002	2.70E-09	32.6
rs7155595	14	77502546	A	C	0.674	-0.013	0.002	2.50E-09	34.4
rs3742365	14	104198251	T	C	0.595	-0.016	0.002	2.50E-14	57.1
rs35175834	15	47680815	G	A	0.788	-0.024	0.002	4.60E-22	89.1
rs28485305	15	74044197	C	T	0.631	0.012	0.002	2.60E-08	31.0
rs8042849	15	78817929	T	C	0.342	0.028	0.002	1.80E-39	163.3
rs8042134	15	97514404	T	G	0.541	-0.014	0.002	1.30E-12	45.0
rs6598539	15	99204483	T	C	0.489	-0.012	0.002	4.50E-09	33.3
rs11861214	16	746611	G	T	0.784	0.014	0.002	2.00E-08	30.7
rs12708665	16	24728227	A	G	0.285	-0.013	0.002	3.50E-09	31.9
rs57611503	16	31165795	G	A	0.485	0.011	0.002	4.00E-08	28.0
rs889398	16	69556715	C	T	0.588	0.013	0.002	6.30E-11	37.9
rs60952428	16	75640521	T	C	0.909	0.019	0.003	3.00E-08	27.6
rs1050847	16	87443734	C	T	0.426	0.011	0.002	1.40E-08	27.4
rs369230	16	89645437	G	T	0.308	-0.013	0.002	1.80E-09	33.3
rs8614	17	27588806	C	A	0.817	-0.017	0.003	1.80E-10	40.0
rs732083	17	37834367	G	A	0.333	0.012	0.002	1.50E-08	29.6
rs67596067	17	50333733	G	A	0.649	-0.013	0.002	1.20E-09	35.6
rs12967855	18	35138245	A	G	0.331	0.012	0.002	3.10E-08	29.5
rs62098013	18	50863861	G	A	0.640	-0.012	0.002	4.10E-09	30.7
rs71367545	18	77576337	G	A	0.791	-0.015	0.002	1.40E-09	34.4
rs76608582	19	4474725	C	A	0.953	0.031	0.005	3.20E-10	39.8
rs35343344	19	18471610	C	A	0.733	0.013	0.002	8.80E-09	30.6
rs4814873	20	19616429	C	T	0.767	0.014	0.002	2.90E-09	32.4
rs6119897	20	31145415	G	A	0.762	-0.018	0.002	3.60E-15	54.4

rs12481282	20	44761377	G	C	0.722	-0.013	0.002	7.80E-09	31.4
rs348809	20	59032097	A	G	0.348	-0.012	0.002	1.30E-08	30.2
rs6011779	20	61984317	C	T	0.191	0.028	0.003	2.30E-27	112.1
rs147412694	21	40702786	G	A	0.850	-0.017	0.003	2.90E-09	34.1
rs2838834	21	46665208	C	T	0.699	-0.013	0.002	6.30E-10	32.9
rs136233	22	31212410	A	G	0.809	-0.014	0.003	1.80E-08	28.0
rs202645	22	41798520	A	G	0.203	-0.015	0.002	3.90E-09	33.7

Abbreviations: SNP, single nucleotide polymorphism; Chr, chromosome; EAF, effect allele frequency; SE, standard error.

Table S3. Result of the first MR analysis of smoking behaviors and cancers (using SNPs as genetic instrumental variables).

Exposure	Outcome	No. of IVs	Method	Beta	SE	P effect	FDR	P heterogeneity	P intercept	P global test
Age of smoking initiation	cervix cancer	9	MR Egger	-1.9035	2.0418	0.382	-	0.571	0.464	-
			Weighted median	-0.0901	0.6005	0.881	-	-	-	-
			Inverse variance weighted	-0.3573	0.4241	0.399	0.599	0.610	-	-
			Simple mode	0.1679	0.9402	0.863	-	-	-	-
			Weighted mode	0.2207	0.8855	0.809	-	-	-	-
			MR-PRESSO	-0.3573	0.3774	0.371	-	-	-	0.550
	endometrial cancer	9	MR Egger	-1.2514	1.7661	0.501	-	0.680	0.647	-
			Weighted median	-0.4198	0.4245	0.323	-	-	-	-
			Inverse variance weighted	-0.4209	0.3318	0.205	0.442	0.751	-	-
			Simple mode	-0.5375	0.6451	0.429	-	-	-	-
			Weighted mode	-0.5829	0.6393	0.388	-	-	-	-
			MR-PRESSO	-0.4209	0.2639	0.149	-	-	-	0.790
	prostate cancer	1	Wald ratio	-0.4278	0.5067	0.398	0.599	-	-	-
	pancreatic cancer	9	MR Egger	4.9554	6.2820	0.456	-	0.792	0.437	-
			Weighted median	-0.4630	1.6496	0.779	-	-	-	-
			Inverse variance weighted	-0.1163	1.2851	0.928	0.944	0.802	-	-
			Simple mode	-1.1068	2.6933	0.692	-	-	-	-
			Weighted mode	-0.7518	2.3837	0.761	-	-	-	-
			MR-PRESSO	-0.1163	0.9719	0.908	-	-	-	0.805

liver cancer	9	MR Egger	0.5043	11.3972	0.966	-	0.133	0.867	-
		Weighted median	-0.2868	2.3554	0.903	-	-	-	-
		Inverse variance weighted	-1.4313	2.1155	0.499	0.696	0.192	-	-
		Simple mode	1.8432	3.2561	0.587	-	-	-	-
		Weighted mode	-0.1013	3.1462	0.975	-	-	-	-
		MR-PRESSO	-1.4313	2.1155	0.518	-	-	-	0.231
testis cancer	9	MR Egger	-0.4740	11.3148	0.968	-	0.599	0.916	-
		Weighted median	2.2981	3.0269	0.448	-	-	-	-
		Inverse variance weighted	0.7364	2.2169	0.740	0.838	0.702	-	-
		Simple mode	4.2510	4.7915	0.401	-	-	-	-
		Weighted mode	4.3110	4.3823	0.354	-	-	-	-
		MR-PRESSO	0.7364	1.8402	0.699	-	-	-	0.700
leukaemia	9	MR Egger	-4.3955	6.2013	0.501	-	0.470	0.380	-
		Weighted median	0.1954	1.5896	0.902	-	-	-	-
		Inverse variance weighted	1.3031	1.2214	0.286	0.505	0.484	-	-
		Simple mode	-0.9517	2.2129	0.678	-	-	-	-
		Weighted mode	-0.0761	2.3298	0.975	-	-	-	-
		MR-PRESSO	1.3031	1.1822	0.302	-	-	-	0.535
multiple myeloma and malignant plasma cell neoplasms	9	MR Egger	5.4243	6.5346	0.434	-	0.487	0.435	-
		Weighted median	1.1222	1.7067	0.511	-	-	-	-
		Inverse variance weighted	0.1178	1.2876	0.927	0.944	0.521	-	-
		Simple mode	1.7660	2.6265	0.520	-	-	-	-

		Weighted mode	1.6845	2.3555	0.495	-	-	-	-
		MR-PRESSO	0.1178	1.2167	0.925	-	-	-	0.560
brain cancer	9	MR Egger	-6.1356	9.3725	0.534	-	0.133	0.593	-
		Weighted median	-2.7284	1.9695	0.166	-	-	-	-
		Inverse variance weighted	-0.9967	1.7613	0.571	0.745	0.168	-	-
		Simple mode	-2.9569	2.7254	0.310	-	-	-	-
		Weighted mode	-3.0246	2.5551	0.271	-	-	-	-
		MR-PRESSO	-0.9967	1.7613	0.587	-	-	-	0.205
biliary cancer	9	MR Egger	8.6658	15.1642	0.586	-	0.510	0.372	-
		Weighted median	-6.3933	4.0611	0.115	-	-	-	-
		Inverse variance weighted	-5.4986	2.9953	0.066	0.264	0.519	-	-
		Simple mode	-9.7592	7.5977	0.235	-	-	-	-
		Weighted mode	-10.7679	6.9317	0.159	-	-	-	-
		MR-PRESSO	-5.4986	2.8341	0.088	-	-	-	0.480
lung cancer	7	MR Egger	-6.7226	2.3029	0.033	-	0.195	0.061	-
		Weighted median	-0.8096	0.6273	0.197	-	-	-	-
		Inverse variance weighted	-1.3262	0.6987	0.058	0.264	0.014	-	-
		Simple mode	-1.1201	0.7779	0.200	-	-	-	-
		Weighted mode	-0.9640	0.7121	0.225	-	-	-	-
		MR-PRESSO	-1.3262	0.6987	0.106	-	-	-	0.020
		MR-PRESSO (Distortion)	-0.5633	0.3588	0.177	-	-	-	<0.005 (1 outlier) [a]
ovary cancer	9	MR Egger	3.0485	1.5169	0.084	-	0.747	0.128	-

		Weighted median	0.5911	0.4051	0.145	-	-	-	-
		Inverse variance weighted	0.4775	0.2878	0.097	0.311	0.509	-	-
		Simple mode	0.6579	0.6402	0.334	-	-	-	-
		Weighted mode	0.7071	0.6199	0.287	-	-	-	-
		MR-PRESSO	0.4775	0.2742	0.120	-	-	-	0.537
rectum cancer	9	MR Egger	-1.0112	3.6775	0.791	-	0.837	0.892	-
		Weighted median	-0.0824	0.8739	0.925	-	-	-	-
		Inverse variance weighted	-0.5012	0.6919	0.469	0.670	0.899	-	-
		Simple mode	0.0138	1.2427	0.991	-	-	-	-
		Weighted mode	-0.0502	1.1520	0.966	-	-	-	-
		MR-PRESSO	-0.5012	0.4581	0.306	-	-	-	0.925
colorectal cancer	9	MR Egger	-1.3712	1.6356	0.430	-	0.678	0.366	-
		Weighted median	-0.0038	0.4144	0.993	-	-	-	-
		Inverse variance weighted	0.1802	0.3161	0.569	0.745	0.671	-	-
		Simple mode	0.0476	0.5903	0.938	-	-	-	-
		Weighted mode	0.0003	0.5762	1.000	-	-	-	-
		MR-PRESSO	0.1802	0.2689	0.522	-	-	-	0.780
breast cancer	9	MR Egger	-1.4414	1.3168	0.310	-	8.68E-04	0.337	-
		Weighted median	-0.1622	0.2074	0.434	-	-	-	-
		Inverse variance weighted	-0.1073	0.2481	0.665	0.778	4.01E-04	-	-
		Simple mode	-0.5299	0.3745	0.195	-	-	-	-
		Weighted mode	-0.3009	0.3127	0.364	-	-	-	-

			MR-PRESSO	-0.1073	0.2481	0.677	-	-	-	0.005
			MR-PRESSO (Distortion)	-0.2623	0.2083	0.248	-	-	-	0.640 (1 outlier) [b]
Smoking initiation	cervix cancer	310	MR Egger	-0.0024	0.2878	0.993	-	0.759	0.167	-
			Weighted median	0.3567	0.0992	3.22E-04	-	-	-	-
			Inverse variance weighted	0.3851	0.0667	7.83E-09	1.57E-07	0.747	-	-
			Simple mode	0.3188	0.2883	0.270	-	-	-	-
			Weighted mode	0.2977	0.2187	0.174	-	-	-	-
			MR-PRESSO	0.3851	0.0649	7.83E-09	-	-	-	0.732
	endometrial cancer	314	MR Egger	-0.1381	0.2359	0.559	-	0.002	0.309	-
			Weighted median	0.0721	0.0790	0.361	-	-	-	-
			Inverse variance weighted	0.0953	0.0565	0.092	0.311	0.002	-	-
			Simple mode	0.4596	0.2958	0.121	-	-	-	-
			Weighted mode	0.3434	0.2633	0.193	-	-	-	-
			MR-PRESSO	0.0953	0.0565	0.093	-	-	-	0.002
	prostate cancer	20	MR Egger	-0.1661	1.2044	0.892	-	3.30E-16	0.749	-
			Weighted median	0.0247	0.1608	0.878	-	-	-	-
			Inverse variance weighted	0.2140	0.2786	0.442	0.647	6.41E-16	-	-
			Simple mode	0.2304	0.2319	0.333	-	-	-	-
			Weighted mode	0.2304	0.2412	0.351	-	-	-	-
			MR-PRESSO	0.2140	0.2786	0.452	-	-	-	<0.002
			MR-PRESSO (Distortion)	0.0507	0.0750	0.508	-	-	-	0.004 (2 outliers) [c]
	pancreatic	311	MR Egger	0.1023	0.8343	0.902	-	0.990	0.874	-

cancer		Weighted median	0.2351	0.2975	0.429	-	-	-	-
		Inverse variance weighted	0.2303	0.2019	0.254	0.492	0.991	-	-
		Simple mode	-0.9323	0.9163	0.310	-	-	-	-
		Weighted mode	-0.8776	0.8588	0.308	-	-	-	-
		MR-PRESSO	0.2303	0.1828	0.209	-	-	-	0.991
liver cancer	299	MR Egger	0.3531	1.1647	0.762	-	0.920	0.581	-
		Weighted median	0.9220	0.4205	0.028	-	-	-	-
		Inverse variance weighted	0.9785	0.2769	4.10E-04	0.004	0.924	-	-
		Simple mode	2.7112	1.2642	0.033	-	-	-	-
		Weighted mode	0.2567	1.1639	0.826	-	-	-	-
		MR-PRESSO	0.9785	0.2605	2.07E-04	-	-	-	0.931
testis cancer	299	MR Egger	-1.7866	1.4398	0.216	-	0.984	0.184	-
		Weighted median	0.1342	0.4920	0.785	-	-	-	-
		Inverse variance weighted	0.0769	0.3420	0.822	0.881	0.983	-	-
		Simple mode	1.3748	1.6040	0.392	-	-	-	-
		Weighted mode	0.7279	1.3678	0.595	-	-	-	-
		MR-PRESSO	0.0769	0.3124	0.806	-	-	-	0.981
leukaemia	299	MR Egger	0.6046	0.7944	0.447	-	0.877	0.376	-
		Weighted median	-0.0802	0.2827	0.777	-	-	-	-
		Inverse variance weighted	-0.0795	0.1887	0.674	0.778	0.878	-	-
		Simple mode	-1.5573	1.0254	0.130	-	-	-	-
		Weighted mode	-1.6650	0.9151	0.070	-	-	-	-

		MR-PRESSO	-0.0795	0.1795	0.658	-	-	-	0.878
multiple myeloma and malignant plasma cell neoplasms	299	MR Egger	1.3136	0.8368	0.118	-	0.517	0.063	-
		Weighted median	-0.4403	0.3135	0.160	-	-	-	-
		Inverse variance weighted	-0.2054	0.1992	0.302	0.518	0.476	-	-
		Simple mode	-0.7545	0.9341	0.420	-	-	-	-
		Weighted mode	-0.4139	0.7866	0.599	-	-	-	-
		MR-PRESSO	-0.2054	0.1992	0.303	-	-	-	0.489
brain cancer	299	MR Egger	0.2983	0.9593	0.756	-	0.395	0.583	-
		Weighted median	-0.2703	0.3281	0.410	-	-	-	-
		Inverse variance weighted	-0.2135	0.2277	0.348	0.568	0.406	-	-
		Simple mode	1.0189	1.2729	0.424	-	-	-	-
		Weighted mode	-1.0269	1.1269	0.363	-	-	-	-
		MR-PRESSO	-0.2135	0.2277	0.349	-	-	-	0.402
biliary cancer	299	MR Egger	-3.6286	2.0222	0.074	-	0.179	0.025	-
		Weighted median	0.5695	0.7267	0.433	-	-	-	-
		Inverse variance weighted	0.7969	0.4834	0.099	0.311	0.137	-	-
		Simple mode	-2.7345	2.3151	0.238	-	-	-	-
		Weighted mode	-0.8326	1.9780	0.674	-	-	-	-
		MR-PRESSO	0.7969	0.4834	0.100	-	-	-	0.139
lung cancer	290	MR Egger	1.0657	0.2881	2.59E-04	-	1.04E-03	0.122	-
		Weighted median	0.6214	0.0959	8.98E-11	-	-	-	-
		Inverse variance weighted	0.6323	0.0692	6.42E-20	3.85E-18	8.28E-04	-	-

		Simple mode	0.8945	0.3410	0.009	-	-	-	-
		Weighted mode	0.9661	0.3664	0.009	-	-	-	-
		MR-PRESSO	0.6323	0.0692	1.16E-17	-	-	-	0.002
		MR-PRESSO (Distortion)	0.6163	0.0683	2.66E-17	-	-	-	0.817(1 outlier) [d]
ovary cancer	305	MR Egger	-0.1166	0.2020	0.564	-	0.013	0.703	-
		Weighted median	-0.0914	0.0677	0.177	-	-	-	-
		Inverse variance weighted	-0.0419	0.0484	0.387	0.599	0.014	-	-
		Simple mode	-0.2021	0.2440	0.408	-	-	-	-
		Weighted mode	-0.2413	0.1864	0.197	-	-	-	-
		MR-PRESSO	0.0419	0.0484	0.387	-	-	-	0.013
		MR-PRESSO (Distortion)	0.0300	0.0475	0.529	-	-	-	0.676 (1 outlier) [e]
rectum cancer	311	MR Egger	-0.4769	0.4485	0.288	-	0.623	0.244	-
		Weighted median	-0.0540	0.1752	0.758	-	-	-	-
		Inverse variance weighted	0.0295	0.1129	0.794	0.881	0.617	-	-
		Simple mode	0.1585	0.5592	0.777	-	-	-	-
		Weighted mode	0.0896	0.4748	0.851	-	-	-	-
		MR-PRESSO	0.0295	0.1115	0.791	-	-	-	0.605
colorectal cancer	312	MR Egger	0.1422	0.2393	0.553	-	1.24E-04	0.719	-
		Weighted median	0.1981	0.0765	0.010	-	-	-	-
		Inverse variance weighted	0.2259	0.0561	5.65E-05	0.001	1.41E-04	-	-
		Simple mode	0.2223	0.2526	0.379	-	-	-	-
		Weighted mode	0.1667	0.2332	0.475	-	-	-	-

		MR-PRESSO	0.2259	0.0561	7.10E-05	-	-	-	1.59E-04
		MR-PRESSO (Distortion)	0.2371	0.0548	2.03E-05	-	-	-	0.839 (1 outlier) [f]
breast cancer	314	MR Egger	0.1161	0.1406	0.410	-	5.87E-55	0.630	-
		Weighted median	0.0400	0.0337	0.235	-	-	-	-
		Inverse variance weighted	0.0503	0.0337	0.135	0.368	7.99E-55	-	-
		Simple mode	0.0091	0.1160	0.938	-	-	-	-
		Weighted mode	0.0091	0.0960	0.925	-	-	-	-
		MR-PRESSO	0.0503	0.0337	0.136	-	-	-	<1.59E-4
		MR-PRESSO (Distortion)	0.0629	0.0262	0.017	-	-	-	0.691 (11 outliers) [g]
cervix cancer	18	MR Egger	-1.2208	0.4013	0.008	-	0.917	0.015	-
		Weighted median	-0.4824	0.2349	0.040	-	-	-	-
		Inverse variance weighted	-0.2245	0.1614	0.164	0.394	0.505	-	-
		Simple mode	-0.5281	0.3783	0.181	-	-	-	-
		Weighted mode	-0.5413	0.2181	0.024	-	-	-	-
		MR-PRESSO	-0.2245	0.1579	0.173	-	-	-	0.460
endometrial cancer	17	MR Egger	-0.4132	0.3660	0.277	-	0.243	0.533	-
		Weighted median	-0.2569	0.1841	0.163	-	-	-	-
		Inverse variance weighted	-0.1968	0.1360	0.148	0.386	0.275	-	-
		Simple mode	0.0225	0.3160	0.944	-	-	-	-
		Weighted mode	-0.1938	0.2318	0.415	-	-	-	-
		MR-PRESSO	-0.1968	0.1360	0.167	-	-	-	0.289
prostate	3	MR Egger	2.0438	1.3961	0.382	-	0.440	0.363	-

cancer		Weighted median	-0.2092	0.2283	0.359	-	-	-	-
		Inverse variance weighted	-0.1143	0.2305	0.620	0.765	0.220	-	-
		Simple mode	-0.3833	0.3444	0.382	-	-	-	-
		Weighted mode	-0.3898	0.3722	0.405	-	-	-	-
		MR-PRESSO	-	-	-	-	-	-	-
pancreatic cancer	18	MR Egger	-0.2129	1.2022	0.862	-	0.720	0.773	-
		Weighted median	0.2079	0.6674	0.755	-	-	-	-
		Inverse variance weighted	0.1125	0.4694	0.811	0.881	0.774	-	-
		Simple mode	-0.0436	1.1218	0.969	-	-	-	-
		Weighted mode	-0.3153	0.8457	0.714	-	-	-	-
		MR-PRESSO	0.1125	0.4013	0.783	-	-	-	0.758
liver cancer	16	MR Egger	-0.7838	2.7577	0.780	-	0.023	0.912	-
		Weighted median	-1.0382	1.0300	0.313	-	-	-	-
		Inverse variance weighted	-1.0721	0.9750	0.272	0.495	0.034	-	-
		Simple mode	-1.4619	1.5326	0.355	-	-	-	-
		Weighted mode	-1.2792	1.1040	0.265	-	-	-	-
		MR-PRESSO	-1.0721	0.9750	0.289	-	-	-	0.065
testis cancer	16	MR Egger	-2.0104	2.6523	0.461	-	0.288	0.393	-
		Weighted median	-0.8129	1.2017	0.499	-	-	-	-
		Inverse variance weighted	0.1617	0.9677	0.867	0.913	0.300	-	-
		Simple mode	-0.6069	1.5515	0.701	-	-	-	-
		Weighted mode	-0.9055	1.3004	0.497	-	-	-	-

		MR-PRESSO	0.1617	0.9677	0.869	-	-	-	0.348
leukaemia	16	MR Egger	-2.4039	1.6251	0.161	-	0.129	0.319	-
		Weighted median	-0.7863	0.6998	0.261	-	-	-	-
		Inverse variance weighted	-0.8427	0.5970	0.158	0.394	0.120	-	-
		Simple mode	0.4381	1.1655	0.712	-	-	-	-
		Weighted mode	-0.4542	0.8524	0.602	-	-	-	-
		MR-PRESSO	-0.8427	0.5970	0.178	-	-	-	0.180
multiple myeloma and malignant plasma cell neoplasms	16	MR Egger	0.8012	1.4321	0.585	-	0.793	0.456	-
		Weighted median	-0.0391	0.7638	0.959	-	-	-	-
		Inverse variance weighted	-0.2213	0.5250	0.673	0.778	0.810	-	-
		Simple mode	0.9597	1.2960	0.470	-	-	-	-
		Weighted mode	0.6965	1.0029	0.498	-	-	-	-
		MR-PRESSO	-0.2213	0.4320	0.616	-	-	-	0.770
brain cancer	16	MR Egger	3.0875	2.2455	0.191	-	0.022	0.223	-
		Weighted median	0.8362	0.8676	0.335	-	-	-	-
		Inverse variance weighted	0.4205	0.8391	0.616	0.765	0.013	-	-
		Simple mode	0.6287	1.4483	0.670	-	-	-	-
		Weighted mode	0.1689	0.9321	0.859	-	-	-	-
		MR-PRESSO	0.4205	0.8391	0.624	-	-	-	0.020
		MR-PRESSO (Distortion)	0.3952	0.6743	0.568	-	-	-	0.965 (2 outliers) [h]
biliary cancer	16	MR Egger	-1.8739	4.0292	0.649	-	0.128	0.237	-
		Weighted median	4.6757	1.6668	0.005	-	-	-	-

		Inverse variance weighted	2.7629	1.4979	0.065	0.264	0.101	-	-
		Simple mode	5.1675	2.4566	0.053	-	-	-	-
		Weighted mode	4.8857	2.0482	0.031	-	-	-	-
		MR-PRESSO	2.7629	1.4979	0.085	-	-	-	0.133
lung cancer	15	MR Egger	1.5973	1.5961	0.335	-	8.51E-23	0.975	-
		Weighted median	0.8807	0.2552	0.001	-	-	-	-
		Inverse variance weighted	1.6445	0.5237	0.002	0.014	2.83E-22	-	-
		Simple mode	0.8317	0.3852	0.049	-	-	-	-
		Weighted mode	0.8949	0.3439	0.021	-	-	-	-
		MR-PRESSO	1.6445	0.5237	0.007	-	-	-	<0.003
		MR-PRESSO (Distortion)	1.1938	0.1544	5.33E-06	-	-	-	<0.003 (2 outliers) [i]
ovary cancer	18	MR Egger	-0.0490	0.2874	0.867	-	0.921	0.499	-
		Weighted median	0.0664	0.1415	0.639	-	-	-	-
		Inverse variance weighted	0.1351	0.1081	0.212	0.442	0.931	-	-
		Simple mode	0.0978	0.2163	0.657	-	-	-	-
		Weighted mode	0.0447	0.1874	0.814	-	-	-	-
		MR-PRESSO	0.1351	0.0800	0.109	-	-	-	0.922
rectum cancer	18	MR Egger	-0.4122	1.1294	0.720	-	0.901	0.969	-
		Weighted median	-0.3665	0.4558	0.421	-	-	-	-
		Inverse variance weighted	-0.3698	0.3370	0.272	0.495	0.931	-	-
		Simple mode	0.1495	0.7294	0.840	-	-	-	-
		Weighted mode	-0.1583	0.6434	0.809	-	-	-	-

			MR-PRESSO	-0.3698	0.2490	0.156	-	-	-	0.924
	colorectal cancer	18	MR Egger	-0.1936	0.3636	0.602	-	0.272	0.530	-
			Weighted median	-0.3771	0.1917	0.049	-	-	-	-
			Inverse variance weighted	-0.4100	0.1345	0.002	0.015	0.304	-	-
			Simple mode	-0.2882	0.3595	0.434	-	-	-	-
			Weighted mode	-0.2469	0.3035	0.427	-	-	-	-
			MR-PRESSO	-0.4100	0.1345	0.007	-	-	-	0.268
	breast cancer	18	MR Egger	0.0731	0.2318	0.756	-	3.55E-05	0.885	-
			Weighted median	0.1539	0.0763	0.044	-	-	-	-
			Inverse variance weighted	0.1047	0.0843	0.215	0.442	6.32E-05	-	-
			Simple mode	0.0536	0.1703	0.757	-	-	-	-
			Weighted mode	0.1250	0.1020	0.237	-	-	-	-
			MR-PRESSO	0.1047	0.0843	0.232	-	-	-	<0.003
			MR-PRESSO (Distortion)	0.1308	0.0766	0.108	-	-	-	0.738
Lifetime smoking index	cervix cancer	125	MR Egger	-0.8446	0.5430	0.122	-	0.247	0.009	-
			Weighted median	0.3364	0.2027	0.097	-	-	-	-
			Inverse variance weighted	0.5413	0.1440	1.71E-04	0.002	0.142	-	-
			Simple mode	0.4532	0.5412	0.404	-	-	-	-
			Weighted mode	0.2501	0.4553	0.584	-	-	-	-
			MR-PRESSO	0.5413	0.1440	2.62E-04	-	-	-	0.145
	endometrial cancer	126	MR Egger	-0.6950	0.4275	0.107	-	0.260	0.021	-
			Weighted median	0.0243	0.1522	0.873	-	-	-	-

		Inverse variance weighted	0.2705	0.1092	0.013	0.071	0.176	-	-
		Simple mode	0.0401	0.4454	0.928	-	-	-	-
		Weighted mode	-0.0983	0.3722	0.792	-	-	-	-
		MR-PRESSO	0.2705	0.1092	0.015	-	-	-	0.166
prostate cancer	7	MR Egger	3.3807	3.4134	0.367	-	0.040	0.426	-
		Weighted median	0.1631	0.3410	0.632	-	-	-	-
		Inverse variance weighted	0.4419	0.3614	0.221	0.442	0.037	-	-
		Simple mode	-0.0683	0.4771	0.891	-	-	-	-
		Weighted mode	-0.0180	0.4863	0.972	-	-	-	-
		MR-PRESSO	0.4419	0.3614	0.267	-	-	-	0.027
		MR-PRESSO (Distortion)	0.0882	0.2473	0.736	-	-	-	<0.007 (1 outlier) [j]
pancreatic cancer	125	MR Egger	-0.1604	1.6169	0.921	-	0.985	0.400	-
		Weighted median	0.8405	0.6043	0.164	-	-	-	-
		Inverse variance weighted	1.1606	0.4085	0.004	0.027	0.985	-	-
		Simple mode	0.9531	1.5275	0.534	-	-	-	-
		Weighted mode	1.3043	1.2493	0.299	-	-	-	-
		MR-PRESSO	1.1606	0.3521	0.001	-	-	-	0.987
liver cancer	124	MR Egger	1.4296	2.4553	0.561	-	0.227	0.858	-
		Weighted median	0.4052	0.8230	0.622	-	-	-	-
		Inverse variance weighted	1.0010	0.5841	0.087	0.311	0.245	-	-
		Simple mode	-0.3590	2.2260	0.872	-	-	-	-
		Weighted mode	-0.2489	1.7668	0.888	-	-	-	-

		MR-PRESSO	1.0010	0.5841	0.089	-	-	-	0.246
testis cancer	124	MR Egger	-3.1749	2.8884	0.274	-	0.906	0.331	-
		Weighted median	-0.0791	0.9925	0.936	-	-	-	-
		Inverse variance weighted	-0.4401	0.6918	0.525	0.716	0.906	-	-
		Simple mode	-1.5475	2.6062	0.554	-	-	-	-
		Weighted mode	-1.9229	2.2435	0.393	-	-	-	-
		MR-PRESSO	-0.4401	0.6327	0.488	-	-	-	0.902
leukaemia	124	MR Egger	0.3455	1.6125	0.831	-	0.424	0.993	-
		Weighted median	0.6460	0.5680	0.255	-	-	-	-
		Inverse variance weighted	0.3586	0.3840	0.350	0.568	0.449	-	-
		Simple mode	1.3752	1.6131	0.396	-	-	-	-
		Weighted mode	1.3752	1.6840	0.416	-	-	-	-
		MR-PRESSO	0.3586	0.3840	0.352	-	-	-	0.446
multiple myeloma and malignant plasma cell neoplasms	124	MR Egger	-1.1182	1.6837	0.508	-	0.895	0.777	-
		Weighted median	-0.6618	0.6163	0.283	-	-	-	-
		Inverse variance weighted	-0.6543	0.4025	0.104	0.311	0.906	-	-
		Simple mode	-0.9458	1.5324	0.538	-	-	-	-
		Weighted mode	-0.6828	1.4282	0.633	-	-	-	-
		MR-PRESSO	-0.6543	0.3682	0.078	-	-	-	0.902
brain cancer	124	MR Egger	3.9905	2.0222	0.051	-	0.172	0.092	-
		Weighted median	0.8023	0.6829	0.240	-	-	-	-
		Inverse variance weighted	0.6536	0.4872	0.180	0.415	0.141	-	-

		Simple mode	0.5793	2.0208	0.775	-	-	-	-
		Weighted mode	0.9425	1.9097	0.623	-	-	-	-
		MR-PRESSO	0.6536	0.4872	0.182	-	-	-	0.147
biliary cancer	124	MR Egger	-1.6989	3.9293	0.666	-	0.538	0.647	-
		Weighted median	0.4483	1.3887	0.747	-	-	-	-
		Inverse variance weighted	0.0548	0.9373	0.953	0.953	0.558	-	-
		Simple mode	-0.1284	3.7702	0.973	-	-	-	-
		Weighted mode	1.4683	3.3171	0.659	-	-	-	-
		MR-PRESSO	0.0548	0.9261	0.953	-	-	-	0.554
lung cancer	107	MR Egger	3.1285	0.7671	8.85E-05	-	1.12E-08	0.025	-
		Weighted median	1.0049	0.2074	1.27E-06	-	-	-	-
		Inverse variance weighted	1.4369	0.1885	2.52E-14	7.56E-13	1.14E-09	-	-
		Simple mode	0.8296	0.5816	0.157	-	-	-	-
		Weighted mode	0.7331	0.5818	0.210	-	-	-	-
		MR-PRESSO	1.4340	0.1811	1.67E-12	-	-	-	<4.16E-4
		MR-PRESSO (Distortion)	1.2575	0.1408	8.44E-15	-	-	-	0.168 (1 outlier) [k]
ovary cancer	105	MR Egger	0.2269	0.3731	0.544	-	0.748	0.981	-
		Weighted median	0.2268	0.1429	0.113	-	-	-	-
		Inverse variance weighted	0.2181	0.0968	0.024	0.120	0.770	-	-
		Simple mode	0.6999	0.3694	0.061	-	-	-	-
		Weighted mode	0.2187	0.3411	0.523	-	-	-	-
		MR-PRESSO	0.2061	0.0867	0.019	-	-	-	0.741

		MR Egger	1.3792	0.9998	0.170	-	0.369	0.196	-
		Weighted median	0.2062	0.3708	0.578	-	-	-	-
		Inverse variance weighted	0.1168	0.2387	0.625	0.765	0.352	-	-
colorectal cancer	126	Simple mode	0.5069	0.8799	0.566	-	-	-	-
		Weighted mode	0.3877	0.7030	0.582	-	-	-	-
		MR-PRESSO	0.1168	0.2387	0.626	-	-	-	0.352
		MR Egger	0.3169	0.4613	0.493	-	0.023	0.945	-
		Weighted median	0.2389	0.1493	0.110	-	-	-	-
		Inverse variance weighted	0.2860	0.1122	0.011	0.066	0.026	-	-
		Simple mode	0.2765	0.4417	0.532	-	-	-	-
		Weighted mode	0.2277	0.3241	0.484	-	-	-	-
		MR-PRESSO	0.2860	0.1122	0.012	-	-	-	0.025
breast cancer	90	MR Egger	0.5070	0.3162	0.112	-	9.99E-11	0.213	-
		Weighted median	0.0999	0.0864	0.248	0.311	-	-	-
		Inverse variance weighted	0.1216	0.0758	0.109	-	5.42E-11	-	-
		Simple mode	-0.2005	0.2565	0.436	-	-	-	-
		Weighted mode	0.0829	0.2019	0.683	-	-	-	-
		MR-PRESSO	0.1216	0.0758	0.112	-	-	-	<5.26E-4
		MR-PRESSO (Distortion)	0.1090	0.0701	0.124	-	-	-	0.844 (3 outliers) [l]

Abbreviations: MR, Mendelian randomization; SNP, single nucleotide polymorphism; IVs, instrumental variables; SE, standard error; FDR, false discovery rate.

Notes: [a,b,d,e,f,j,k] The P value was provided by the distortion test, with 1 outlier observed; [c,h,j] The P value was provided by the distortion test, with 2 outliers observed; [l] The P value was provided by the distortion test, with 3 outliers observed; [g] The P value was provided by the distortion test, with 11 outliers observed.

Table S4. Details of significant associations (FDR<0.05) between smoking-related aberrant CPG sites and cancers.

Cancer	CpG site	Chr	Position	related-gene	SNPs	Inverse variance weighted			
						Beta (adjusted)	SE	P value	FDR
Biliary cancer	cg10130088	11	122138618	-	3	1.6525	0.4312	1.27E-04	2.12E-02
Breast cancer	cg00395063	11	120210293	ARHGEF12	1	0.1545	0.0434	3.71E-04	2.30E-02
	cg02068690	2	25600451	DTNB	5	0.0654	0.0184	3.74E-04	3.87E-02
	cg02145310	10	80844809	ZMIZ1	1	0.0521	0.0119	1.23E-05	1.71E-03
	cg02405476	20	44441818	UBE2C	2	0.0540	0.0147	2.41E-04	1.82E-02
	cg03140135	12	13350820	EMP1	7	0.0389	0.0095	4.20E-05	8.16E-03
	cg04209460	17	4711018	PLD2/RP11-81A22.5	5	0.0287	0.0088	1.04E-03	4.09E-02
	cg04304450	22	43525431	BIK	2	0.1171	0.0328	3.55E-04	2.37E-02
	cg04521626	17	4714200	PLD2	2	0.0962	0.0237	4.80E-05	5.43E-03
	cg04551776	5	393366	AHRR	2	0.1050	0.0300	4.70E-04	1.96E-02
	cg05318210	2	145780010	AC074093.1	3	0.0652	0.0185	4.34E-04	3.91E-02
	cg06639488	1	155103222	EFNA1	1	0.2043	0.0434	2.48E-06	1.12E-03
	cg07465627	17	53167407	STXBP4	1	0.0782	0.0181	1.58E-05	4.93E-03
	cg07932199	12	112008034	ATXN2	1	0.3129	0.0596	1.52E-07	6.38E-05
	cg08022502	15	91479023	HDDC3/UNC45A	1	0.0902	0.0173	1.78E-07	3.71E-05
	cg08260891	20	44518802	CTSA/NEURL2	1	0.1120	0.0254	1.01E-05	2.28E-03
	cg10666909	6	32820249	PSMB9/TAP1	1	0.2042	0.0558	2.56E-04	2.37E-02
	cg10951873	1	25254746	RUNX3	1	0.1111	0.0323	5.95E-04	4.55E-02

	cg11152384	11	68934300	RP11-554A11.8	1	0.2371	0.0589	5.66E-05	1.26E-02
	cg11305121	2	121011590	RALB	3	0.0649	0.0127	3.35E-07	5.63E-05
	cg12101586	15	75019203	CYP1A1	1	0.1531	0.0340	6.66E-06	1.39E-03
	cg14142171	6	30228101	HLA-L	5	0.0606	0.0161	1.67E-04	1.44E-02
	cg14391586	20	62681296	SOX18	5	0.0375	0.0110	6.22E-04	3.21E-02
	cg14817490	5	392920	AHRR	2	0.0684	0.0159	1.71E-05	6.03E-03
	cg15963463	1	25253237	RUNX3	1	0.1322	0.0386	6.07E-04	4.01E-02
	cg16779839	17	79374327	BAHCC1	2	0.0449	0.0141	1.40E-03	4.23E-02
	cg17065712	10	80850605	ZMIZ1	1	0.3596	0.0422	1.68E-17	6.97E-15
	cg17777683	2	201986522	CFLAR	2	0.1297	0.0351	2.19E-04	2.37E-02
	cg20366110	8	124720897	ANXA13	1	0.3266	0.0470	3.82E-12	1.73E-09
	cg21161138	5	399360	AHRR	1	0.1908	0.0583	1.07E-03	3.35E-02
	cg25004427	5	326523	AHRR/PDCD6	2	0.2337	0.0415	1.84E-08	7.97E-06
	cg25727671	7	27193351	HOXA-AS3/HOXA3/HOXA7/RP1-170O19.22/RP1-170O19.23	2	0.1292	0.0360	3.35E-04	2.37E-02
	cg25839482	15	75931953	IMP3/CTD-2026K11.4	4	0.0530	0.0097	5.30E-08	2.47E-05
	cg26954197	5	436816	AHRR	1	0.1139	0.0287	7.10E-05	1.25E-02
Cervix cancer	cg07525886	17	8227927	-	1	0.2935	0.0786	1.89E-04	3.00E-02
	cg14142171	6	30228101	HLA-L	5	0.1174	0.0340	5.63E-04	3.00E-02
	cg19758448	17	37828296	PGAP3	4	0.1002	0.0275	2.67E-04	4.46E-02
	cg24033122	16	30485383	ITGAL	9	0.1226	0.0291	2.61E-05	5.05E-03
Colorectal cancer	cg07932199	12	112008034	ATXN2	1	0.8960	0.1453	7.01E-10	1.37E-06
	cg17823346	10	80848143	ZMIZ1	5	0.1222	0.0275	8.97E-06	5.85E-03

Endometrial cancer	cg03531211	6	32920102	HLA-DMA	5	0.1777	0.0406	1.17E-05	4.08E-03
	cg05222924	11	32450486	WT1	2	0.2788	0.0650	1.79E-05	7.01E-03
	cg07932199	12	112008034	ATXN2	1	0.9757	0.1517	1.27E-10	5.39E-08
	cg21769619	11	32454768	WT1	1	0.3957	0.0859	4.15E-06	1.80E-03
Liver cancer	cg01765406	2	129231478	-	1	2.9051	0.7970	2.67E-04	4.52E-02
	cg27424326	2	129251303	-	1	1.1134	0.3054	2.67E-04	4.46E-02
Lung cancer	cg06639488	1	155103222	EFNA1	1	0.5158	0.1398	2.23E-04	1.76E-02
	cg06972908	6	30488321	ITGAL	1	0.7642	0.2226	5.98E-04	4.67E-02
	cg07932199	12	112008034	ATXN2	1	0.6535	0.1754	1.95E-04	3.04E-02
	cg18419271	6	32116016	-	2	0.3865	0.0892	1.46E-05	6.02E-03
	cg20069688	6	31941049	DXO	6	0.2520	0.0613	3.92E-05	5.91E-03
	cg22561727	19	4719848	DPP9	1	0.8181	0.2372	5.62E-04	2.96E-02
	cg23320649	3	50604613	C3orf18	1	0.4715	0.1396	7.29E-04	4.41E-02
Ovarian cancer	cg03333699	7	966569	ADAP1/COX19	3	0.2158	0.0401	7.47E-08	3.61E-05
	cg11145461	1	201475451	CSRP1	1	0.2266	0.0592	1.30E-04	1.00E-02
	cg11375458	2	177003996	HOXD3	1	0.2692	0.0689	9.25E-05	4.02E-02
	cg22533573	11	32452771	WT1	2	0.3890	0.1024	1.45E-04	2.29E-02
Prostate cancer	cg00867472	1	156714808	HDGF	1	0.3394	0.0923	2.35E-04	9.73E-03
	cg02405476	20	44441818	UBE2C	2	0.0651	0.0185	4.35E-04	1.38E-02
	cg03665259	6	31021826	XXbac-BPG118E17.6	2	0.3525	0.0566	4.65E-10	5.41E-08
	cg03812323	6	32116106	-	2	0.1297	0.0366	3.94E-04	1.38E-02
	cg05394800	13	50707050	DLEU1	2	0.2565	0.0588	1.29E-05	1.53E-03

cg06857018	7	1908314	MAD1L1	1	0.3071	0.0824	1.94E-04	9.73E-03
cg08899667	6	31761055	VARS	2	0.1896	0.0578	1.04E-03	2.99E-02
cg10255761	3	49210029	KLHDC8B	1	0.0672	0.0176	1.38E-04	9.56E-03
cg11152384	11	68934300	RP11-554A11.8	1	0.9469	0.0979	3.86E-22	9.15E-20
cg12101586	15	75019203	CYP1A1	1	0.1401	0.0453	2.00E-03	4.39E-02
cg12406027	19	39616818	PAK4	1	0.1876	0.0591	1.51E-03	4.15E-02
cg12593793	1	156074135	LMNA	1	0.2342	0.0610	1.23E-04	9.73E-03
cg13782301	6	32116875	PRRT1	4	0.0886	0.0245	3.07E-04	1.02E-02
cg14258501	6	31680228	LY6G6E/XXbac-BPG32J3.19/XXbac-BPG32J3.20	1	0.2936	0.0468	3.65E-10	8.14E-08
cg14757228	6	32116858	PRRT1	3	0.1582	0.0373	2.25E-05	1.67E-03
cg15948030	6	31760825	VARS	2	0.2469	0.0579	2.03E-05	1.48E-03
cg16274678	1	154127952	NUP210L/TPM3	2	0.0500	0.0146	6.46E-04	2.23E-02
cg22533573	11	32452771	WT1	2	0.2317	0.0623	2.01E-04	1.26E-02
cg22740603	6	33397959	SYNGAP1	2	0.0560	0.0148	1.48E-04	1.52E-02
cg23061027	6	32116207	PRRT1	4	0.1157	0.0313	2.22E-04	9.58E-03
cg25727671	7	27193351	HOXA-AS3/HOXA3/HOXA7/RP1-170O19.22/RP1-170O19.23	1	0.2346	0.0677	5.34E-04	3.60E-02
cg25955180	6	32116538	PRRT1	5	0.1007	0.0271	2.06E-04	9.58E-03
cg26146569	15	31637592	KLF13	1	0.1000	0.0270	2.12E-04	3.35E-02
cg26203136	7	739057	PRKAR1B	2	0.0965	0.0321	2.63E-03	4.88E-02
cg26257411	6	33397122	SYNGAP1	4	0.0617	0.0144	1.85E-05	1.60E-03
cg26844633	15	40635904	-	1	0.2305	0.0675	6.39E-04	2.48E-02

	cg27514333	15	66996626	SMAD6	2	0.0788	0.0247	1.43E-03	3.80E-02
Rectum cancer	cg18091264	15	66795595	SNORD18A;RPL4;SNORD18B;SNORD16	1	0.4197	0.1170	3.35E-04	4.11E-02

Abbreviations: FDR, false discovery rate; Chr, chromosome; SNP, single nucleotide polymorphism, SE, standard error.

Table S5. MQTLs of CpG sites overlapped with expression.

CpG sites	Gene symbol	Cancer	mQTLs	Tissue-specific association	
				p-value	m-value
cg06639488	EFNA1	breast cancer	rs11264328	6.00E-03	0.97
		lung cancer	rs9330263	2.10E-05	1.00
cg12101586	CYP1A1	breast cancer	rs2472299	4.60E-05	1.00
cg14142171	HLA-L	breast cancer	rs1611463	5.00E-06	1.00
cg22533573	WT1	prostate cancer	rs2301250	0.10	0.76
cg25727671	HOXA7	prostate cancer	rs1801085	0.70	0.17
		breast cancer	rs1801085	0.40	0.60
			rs62454419	1.00	0.02

Abbreviations: mQTLs, methylation quantitative trait loci.

Table S6. Colocalization analysis of CpG sites significantly associated with multiple cancers.

Cancer	CpG sites	related-gene	SNPs (mQTLs)	Summary.PP.H4	SNP.PP.H4	Notes
Breast cancer	cg04521626	PLD2	rs2241933	88.0%	100.0%	-
	cg06639488	EFNA1	rs12137164	91.6%	89.3%	-
	cg07932199	ATXN2	rs7310615	98.9%	99.8%	with only 9 SNPs
	cg10666909	PSMB9/TAP1	rs2071542	82.9%	99.1%	-
	cg11152384	RP11-554A11.8	rs12796066	90.7%	99.8%	with only 16 SNPs
	cg20366110	ANXA13	rs7014939	98.6%	94.6%	-
	cg25839482	IMP3/CTD-2026K11.4	rs12708519	85.5%	89.7%	-
Colorectal cancer	cg07932199	ATXN2	rs7310615	98.8%	99.2%	with only 9 SNPs
Endometrial cancer	cg03531211	HLA-DMA	rs9276731	94.8%	99.9%	-
	cg07932199	ATXN2	rs7310615	99.7%	99.5%	with only 9 SNPs
Liver cancer	cg01765406	-	rs841080	91.4%	100.0%	with only 30 SNPs
	cg27424326	-	rs841080	91.2%	100.0%	-
Lung cancer	cg07932199	ATXN2	rs3184504	88.7%	98.5%	with only 6 SNPs
	cg20069688	DXO	rs433061	76.7%	99.8%	-
	cg22561727	DPP9	rs16992471	85.2%	100.0%	with only 2 SNPs
prostate cancer	cg00867472	HDGF	rs12140437	76.2%	100.0%	with only 2 SNPs
	cg12593793	LMNA	rs10047112	86.2%	100.0%	with only 1 SNP
	cg26146569	KLF13	rs4779862	78.0%	100.0%	with only 4 SNPs

Abbreviations: FDR, false discovery rate; SNPs, single nucleotide polymorphisms; mQTLs, methylation quantitative trait loci; PP, posterior probability.

Supplementary figures

Figure S1. Heatmap of CpG sites with cross-cancer effect.

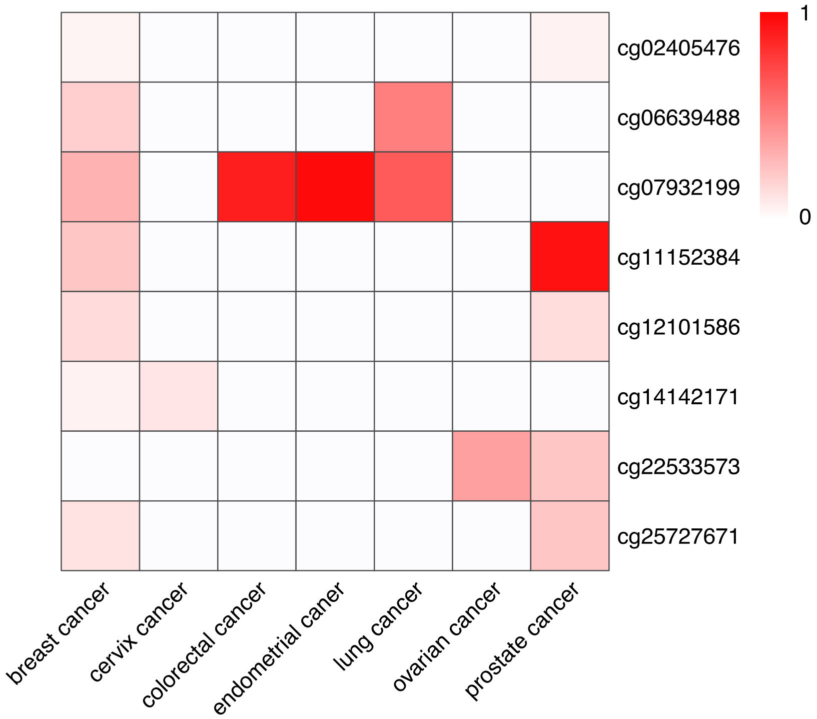

Figure S2. Colocalization evidence for methylation at CpG site cg04521616 and susceptibility to breast cancer.

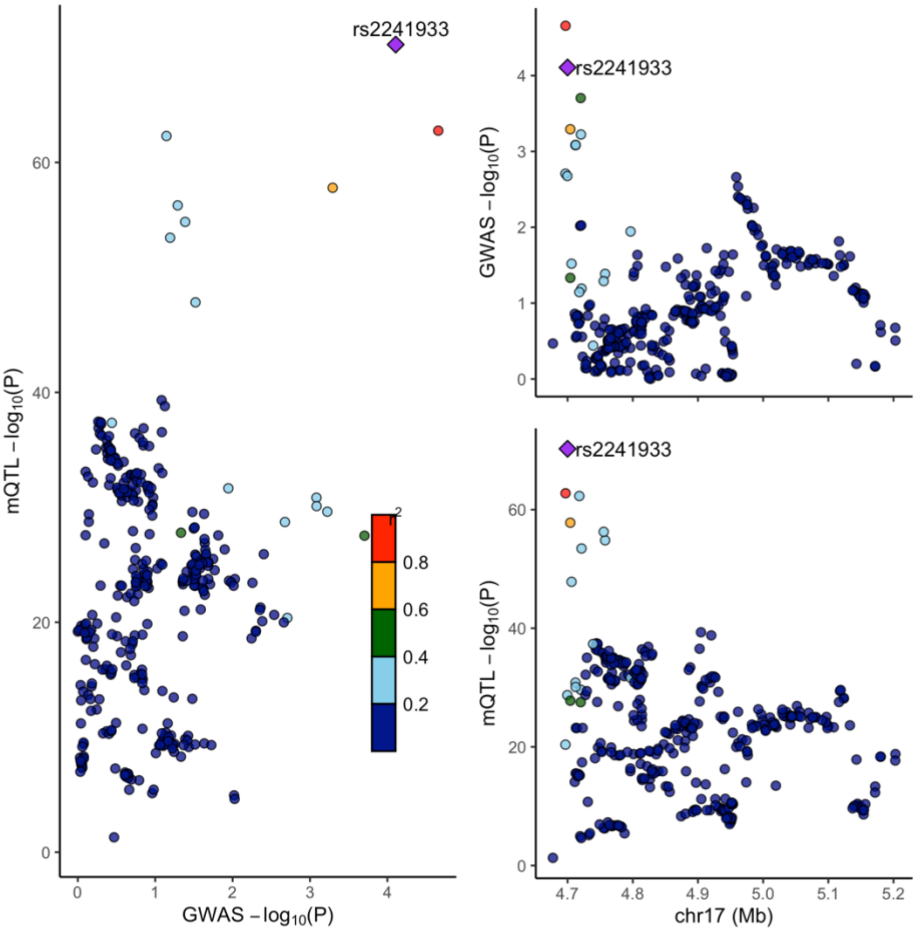

Abbreviations: mQTL, methylation quantitative trait loci; P, p-value; GWAS, genome-wide association study; chr, chromosome.

Figure S3. Colocalization evidence for methylation at CpG site cg06639488 and susceptibility to breast cancer.

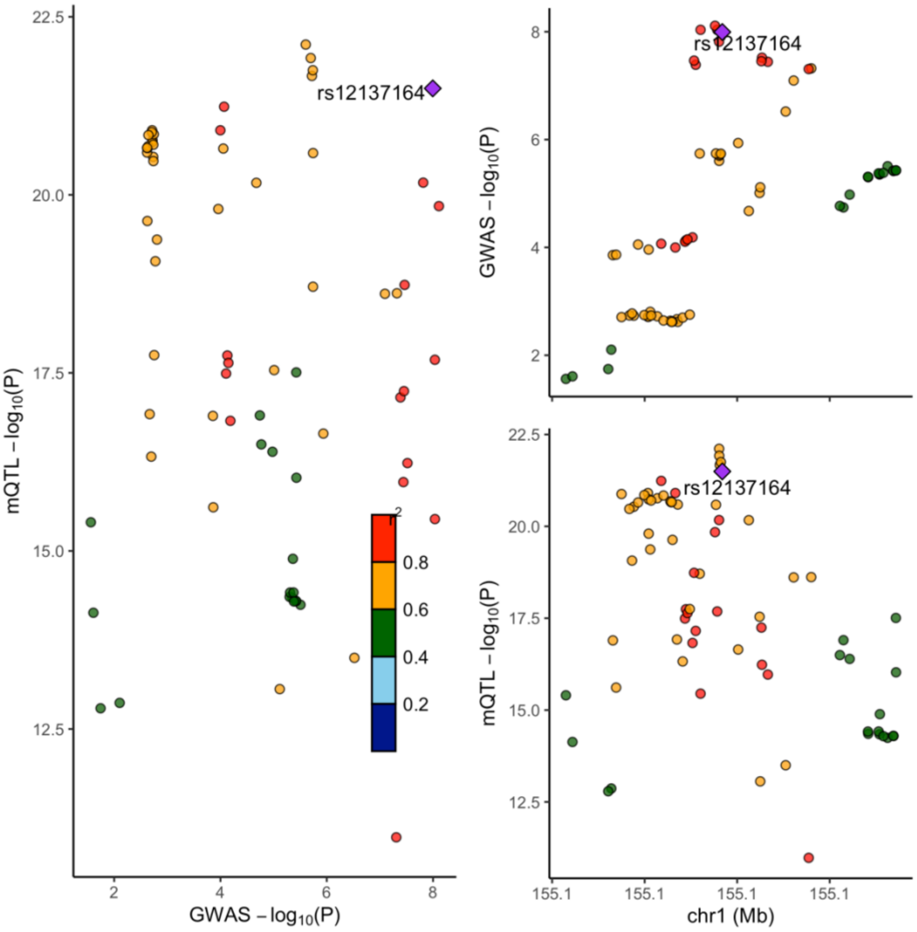

Abbreviations: mQTL, methylation quantitative trait loci; P, p-value; GWAS, genome-wide association study; chr, chromosome.

Figure S4. Colocalization evidence for methylation at CpG site cg10666909 and susceptibility to breast cancer.

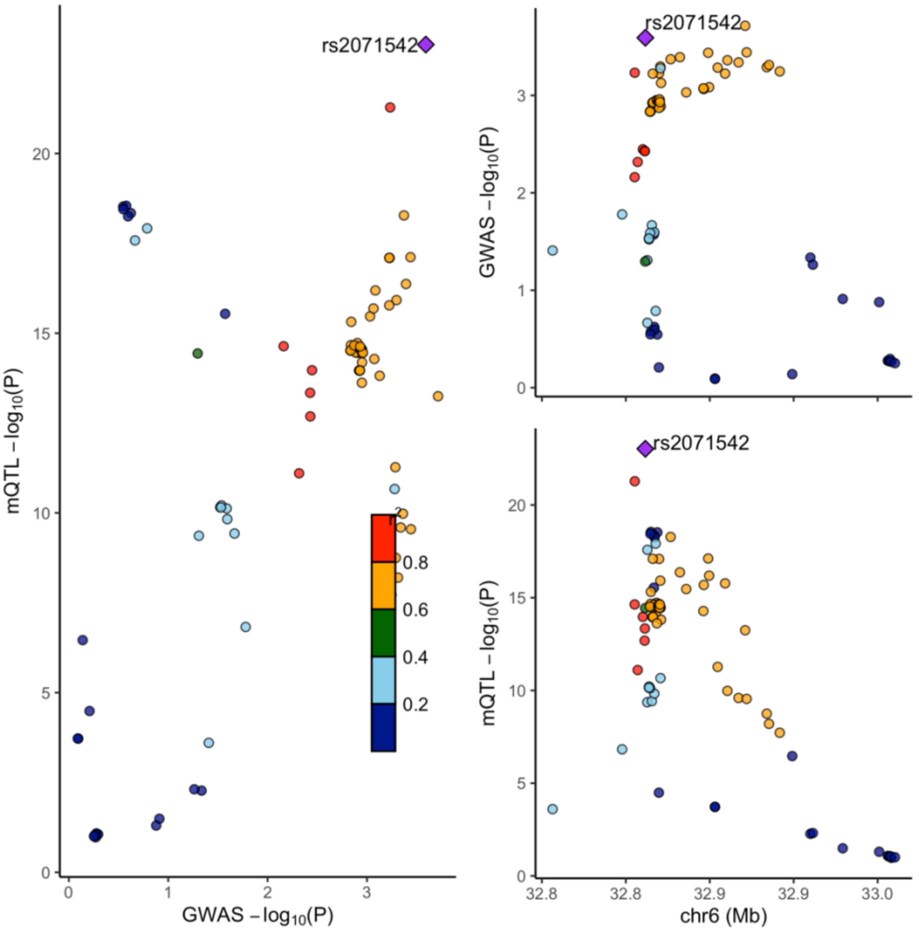

Abbreviations: mQTL, methylation quantitative trait loci; P, p-value; GWAS, genome-wide association study; chr, chromosome.

Figure S5. Colocalization evidence for methylation at CpG site cg20366110 and susceptibility to breast cancer.

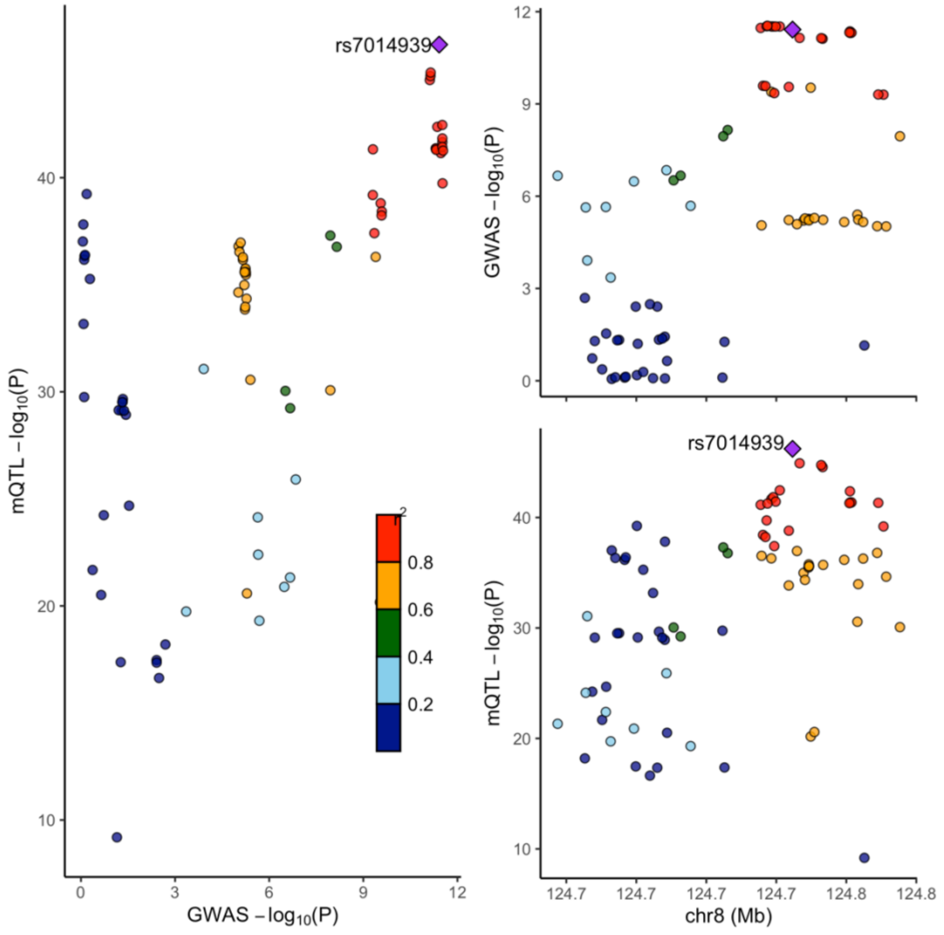

Abbreviations: mQTL, methylation quantitative trait loci; P, p-value; GWAS, genome-wide association study; chr, chromosome.

Figure S6. Colocalization evidence for methylation at CpG site cg25839482 and susceptibility to breast cancer.

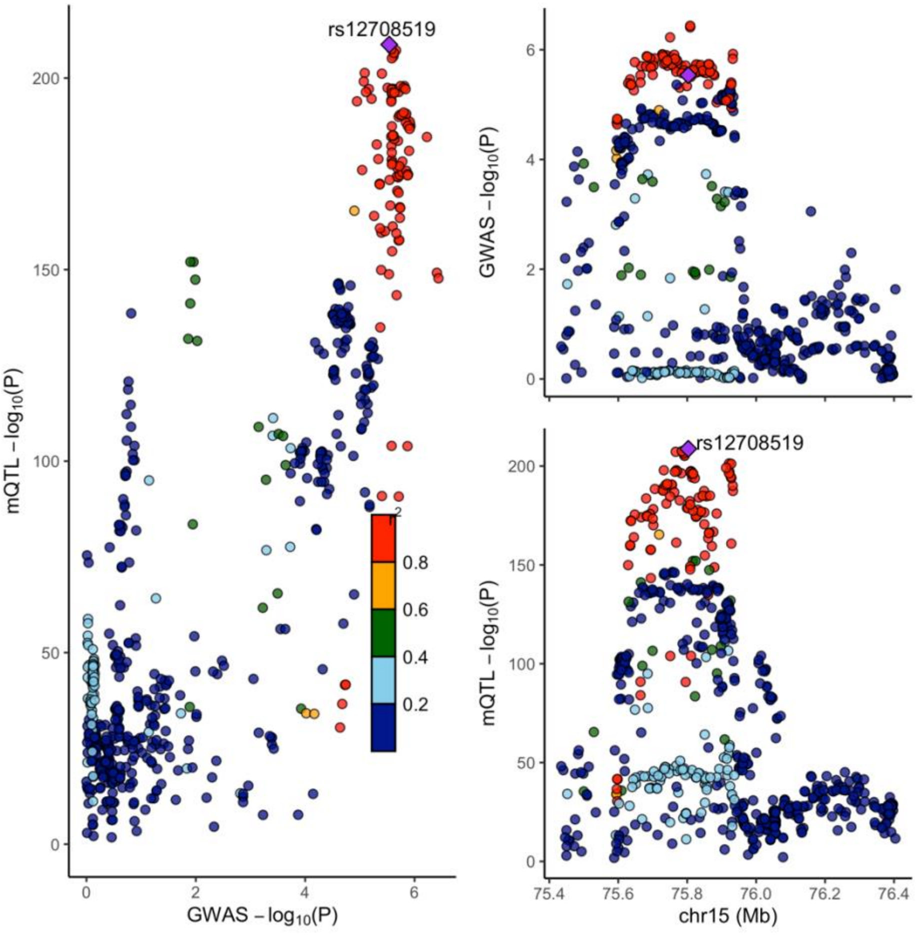

Abbreviations: mQTL, methylation quantitative trait loci; P, p-value; GWAS, genome-wide association study; chr, chromosome.

Figure S7. Colocalization evidence for methylation at CpG site cg03531211 and susceptibility to endometrial cancer.

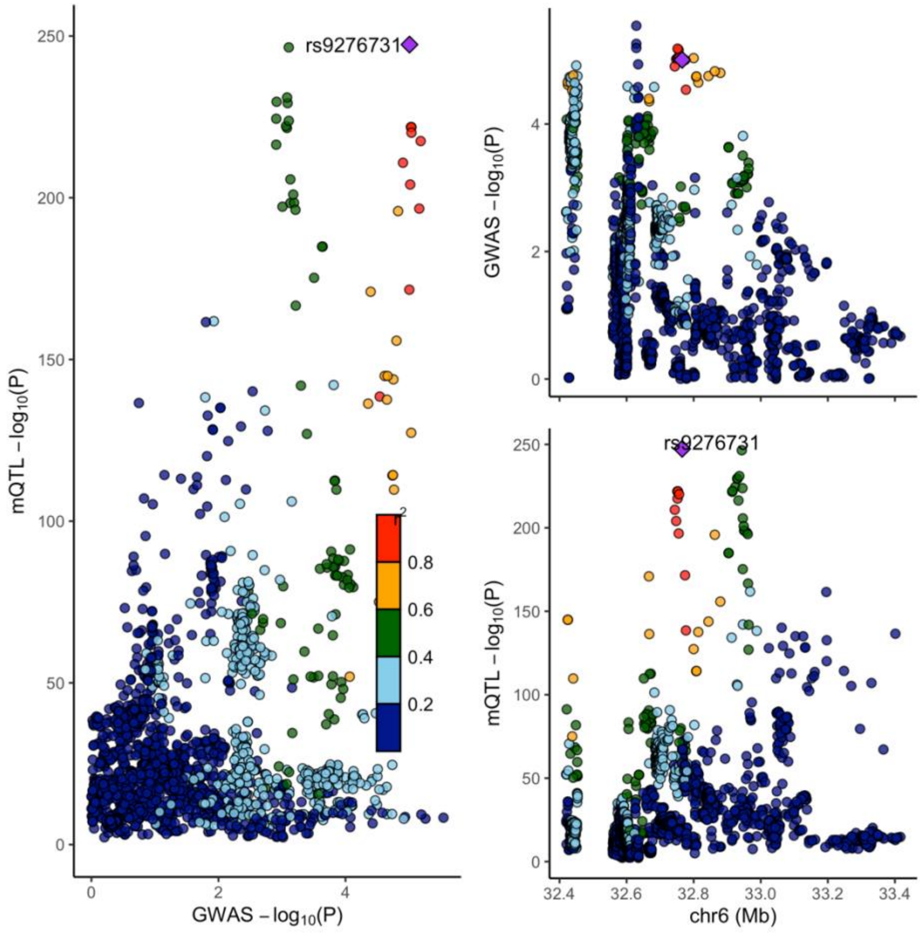

Abbreviations: mQTL, methylation quantitative trait loci; P, p-value; GWAS, genome-wide association study; chr, chromosome.

Figure S8. Colocalization evidence for methylation at CpG site cg27424326 and susceptibility to liver cancer.

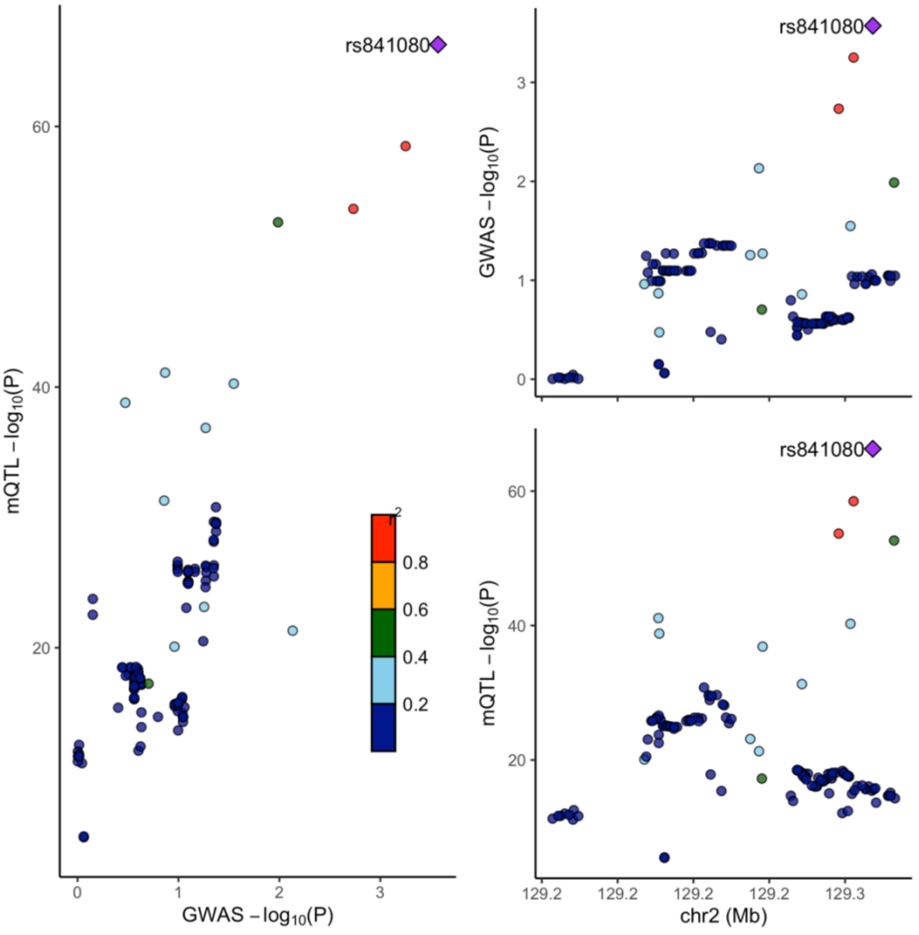

Abbreviations: mQTL, methylation quantitative trait loci; P, p-value; GWAS, genome-wide association study; chr, chromosome.

Figure S9. Colocalization evidence for methylation at CpG site cg20069688 and susceptibility to lung cancer.

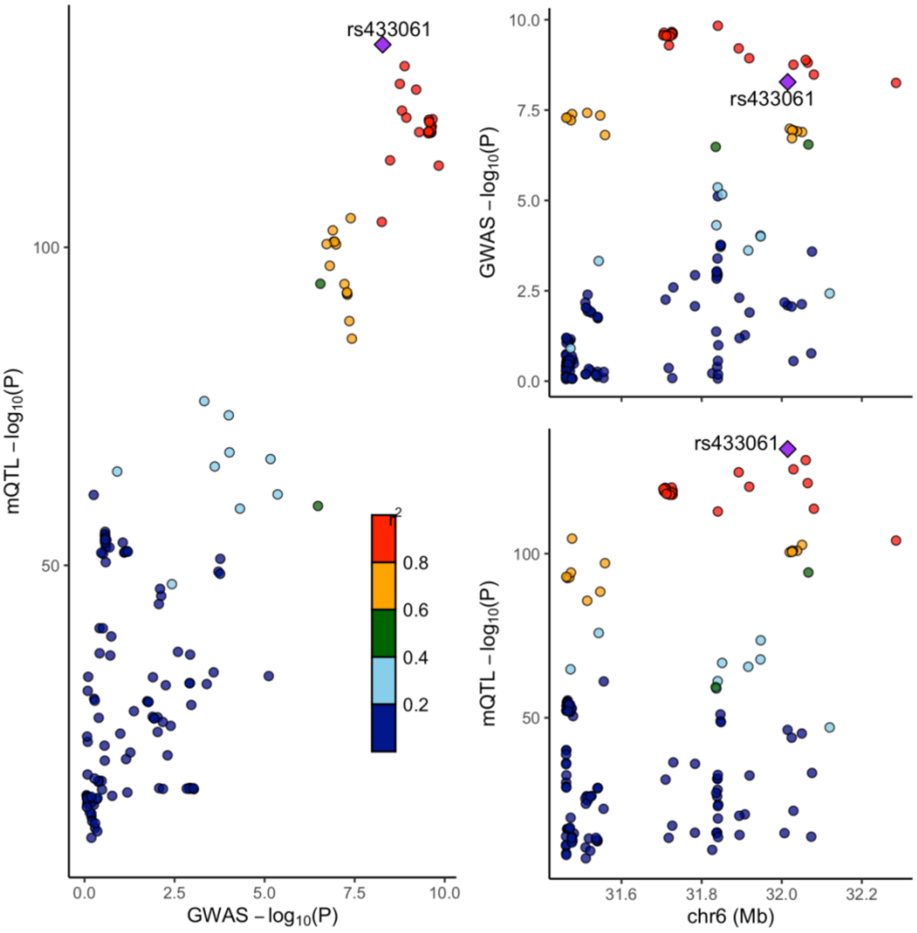

Abbreviations: mQTL, methylation quantitative trait loci; P, p-value; GWAS, genome-wide association study; chr, chromosome.
